# Medication-related interventions to improve medication safety and patient outcomes on transition from adult intensive care settings: a systematic review and meta-analysis

**DOI:** 10.1136/bmjqs-2021-013760

**Published:** 2022-01-18

**Authors:** Richard S Bourne, Jennifer K Jennings, Maria Panagioti, Alexander Hodkinson, Anthea Sutton, Darren M Ashcroft

**Affiliations:** 1 Pharmacy and Critical Care, Sheffield Teaching Hospitals NHS Foundation Trust, Sheffield, UK; 2 School of Health Sciences, Faculty of Biology, Medicine and Health, The University of Manchester, Manchester, UK; 3 National Institute for Health Research (NIHR) Greater Manchester Patient Safety Translational Research Centre (PSTRC), School of Health Sciences, Faculty of Biology, Medicine and Health, The University of Manchester, Manchester, UK; 4 National Institute for Health Research (NIHR) School for Primary Care Research, Division of Population Health, Health Services Research and Primary Care, School of Health Sciences, Faculty of Biology, Medicine and Health, The University of Manchester, Manchester, UK; 5 School of Health and Related Sciences (ScHARR), The University of Sheffield, Sheffield, Sheffield, UK

**Keywords:** critical care, medication safety, patient safety, transitions in care

## Abstract

**Background:**

Patients recovering from an episode in an intensive care unit (ICU) frequently experience medication errors on transition to the hospital ward. Structured handover recommendations often underestimate the challenges and complexity of ICU patient transitions. For adult ICU patients transitioning to a hospital ward, it is currently unclear what interventions reduce the risks of medication errors.

The aims were to examine the impact of medication-related interventions on medication and patient outcomes on transition from adult ICU settings and identify barriers and facilitators to implementation.

**Methods:**

The systematic review protocol was preregistered on PROSPERO. Six electronic databases were searched until October 2020 for controlled and uncontrolled study designs that reported medication-related (ie, de-prescribing; medication errors) or patient-related outcomes (ie, mortality; length of stay). Risk of bias (RoB) assessment used V.2.0 and ROBINS-I Cochrane tools. Where feasible, random-effects meta-analysis was used for pooling the OR across studies. The quality of evidence was assessed by Grading of Recommendations, Assessment, Development and Evaluations.

**Results:**

Seventeen studies were eligible, 15 (88%) were uncontrolled before-after studies. The intervention components included education of staff (n=8 studies), medication review (n=7), guidelines (n=6), electronic transfer/handover tool or letter (n=4) and medicines reconciliation (n=4). Overall, pooled analysis of all interventions reduced risk of inappropriate medication continuation at ICU discharge (OR=0.45 (95% CI 0.31 to 0.63), I^2^=55%, n=9) and hospital discharge (OR=0.39 (95% CI 0.2 to 0.76), I^2^=75%, n=9). Multicomponent interventions, based on education of staff and guidelines, demonstrated no significant difference in inappropriate medication continuation at the ICU discharge point (OR 0.5 (95% CI 0.22 to 1.11), I^2^=62%, n=4), but were very effective in increasing de-prescribing outcomes on hospital discharge (OR 0.26 (95% CI 0.13 to 0.55), I^2^=67%, n=6)). Facilitators to intervention delivery included ICU clinical pharmacist availability and participation in multiprofessional ward rounds, while barriers included increased workload associated with the discharge intervention process.

**Conclusions:**

Multicomponent interventions based on education of staff and guidelines were effective at achieving almost four times more de-prescribing of inappropriate medication by the time of patient hospital discharge. Based on the findings, practice and policy recommendations are made and guidance is provided on the need for, and design of theory informed interventions in this area, including the requirement for process and economic evaluations.

## Background

Delivering quality and safety at patient care transitions is challenging and complex,^1^ with patient outcomes affected by a wide range of interacting system and process components.^2^ Medication safety in transitions of patient care is a key priority area for the third WHO’s Global Patient Safety Challenge—‘medication without harm’.^3 4^

For adult patients surviving an intensive care unit (ICU) care episode, the transition to a hospital ward is especially challenging for care continuity and safety.^5 6^ ICU patients may experience a protracted recovery, further compounded by polypharmacy and care fragmentation.^7^ They also encounter frequent medication changes, with many chronic medicines discontinued and acute medication commenced,^8–10^ presenting a patient safety concern,^11^ particularly at the point of transitions.^12^

Medication errors (MEs) are common in adult ICU patients at the interface of transfer to the hospital ward, with MEs reported to occur in between 46% and 74% of patients.^13–15^ The most commonly reported types of ME involve continuation of potentially inappropriate medication, discontinuation of important chronic medication and inappropriate dose or route of administration.^13–15^ The reported incidence of adverse drug events (ADEs) related to ICU patient transfer varies from 6% to 70%,^13 15 16^ according to surveillance period and methodology employed, being highest in the intervention study with prospectively collected data.^13^ MEs post-ICU care can also continue long after patient hospital discharge.^17–20^ Pre-existing polypharmacy burden may predict ME risk,^21^ and the risk of unplanned hospital readmission.^22^

To mitigate this increased patient safety risk, the European Society of Intensive Care Medicine recommends use of a standardised handover procedure, including an explanation of medication changes and treatment plans, on patient transfer from ICU.^23^ However, ICU medication-related discharge practices remain inconsistent.^24–27^ Contributors to variation in practice include uncertainty over key intervention components and processes,^24^ availability of important medicines optimisation resources^25^ and staff communication failures^26^ in the context of an important care interface.^6^ Moreover, in isolation, the recommended handover procedure does not adequately address the challenges and complexity of patient care transitions including the importance of patient and family engagement, medicines reconciliation and medication review.^1 2 4 6 28^

A recent systematic review demonstrated the benefits of pharmacy-led interventions to improve de-prescribing of stress ulceration prophylaxis (SUP) at ICU and hospital discharge points.^29^ However, a broader scope and multiprofessional approach is required to meet the wider medicines optimisation and communication challenges to transitional medication continuity and safety.^4^ For ICU patient to hospital ward transitions, it is unclear what medication-related intervention components are required and their efficacy, timing and mode of delivery. This information is required to help optimise existing medicines-related practice for ICU patients on transition to a hospital ward, and aid identification of evidence gaps, informing the need and design of further research to improve patient safety.

This systematic review and meta-analysis aimed to (i) examine the impact of medication-related interventions on medication and patient outcomes on transition from adult ICU settings and (ii) identify barriers and facilitators during intervention implementation.

## Methods

We performed a systematic review and meta-analysis according to the Preferred Reporting Items for Systematic Reviews and Meta-Analyses (PRISMA) 2020 statement.^30^ The protocol was preregistered on the international prospective register of systematic reviews (PROSPERO) database (CRD42020210638). Patient and public involvement and engagement was provided by representatives from intensive care and emergency care forums in the scope of review and planned dissemination of results.

### Eligibility criteria

We included studies conducted in adult (≥18 years old) ICU patients surviving to transition to a hospital ward, investigating any medication-related interventions designed to affect medication continuity, safety or efficacy for ICU patients transitioning to a hospital ward, compared with usual care; that is, a non-exposed control group or historical group in a before-after study, evaluating medication-related (eg, MEs) or patient-related outcomes (eg, length of stay, mortality). Interventions had to be conducted before or within 48 hours of ICU patient transition to the hospital ward. We used the Cochrane Effective Practice and Organisation of Care Group criteria as a guide for study eligibility.^31^ Randomised controlled trials (RCTs) and non-randomised controlled and uncontrolled trials (including ‘before-after’ and ‘interrupted time series’ designs) were eligible for inclusion. Review articles, studies based on simulation and conference abstracts were excluded.

### Information sources

In October 2020, the following databases were searched from inception without language restrictions: MEDLINE and MEDLINE Epub Ahead of Print, In-Process and Other Non-Indexed Citations and Daily; EMBASE; CINAHL; International Pharmaceutical Abstracts; The Cochrane Library; Science Citation Index. A search of the trial registries International Clinical Trials Registry Platform and ClinicalTrials.gov was conducted in August 2021 (online supplemental file 1).

10.1136/bmjqs-2021-013760.supp1 Supplementary data

### Selection process

Two independent reviewers (RSB, JKJ) participated in each phase of the selection process. Any disagreement between reviewers was resolved by discussion, with arbitration by a third reviewer (DMA) when required. First, titles and abstracts were screened based on our eligibility criteria. Second, full texts were screened. Finally, reference lists of included studies and any relevant reviews were checked for any further relevant references. The citation search facility in Web of Science was used to identify any relevant cited references and additional studies by key authors. Authors of all eligible abstracts published within the last 2 years were contacted to confirm recent or planned publication.^32–36^

### Data extraction

Two reviewers (RSB, JKJ) independently extracted data in duplicate from included studies using a standardised data extraction form (with cross-checking validation process). The structured data collection form was prepiloted, and data inputted into a proprietary software database (*Covidence*; www.covidence.org).

### Risk of bias in individual studies

Quality assessment of individual studies was done by two independent authors (RSB, JKJ) using the Cochrane risk of bias (RoB) V.2.0 tool for RCTs,^37^ and ROBINS-I for non-randomised studies.^38^ Any disagreement was resolved by discussion, with arbitration by a third independent reviewer (MP) when required. RoB judgements are presented using the *robvis* visualisation tool (www.riskofbias.info/welcome/robvis-visualization-tool). Studies with a ‘high’ or ‘critical’ RoB assessment^37 38^ were excluded from any meta-analysis.

### Confidence in overall evidence using Grading of Recommendations, Assessment, Development and Evaluations

We undertook a Grading of Recommendations, Assessment, Development and Evaluations (GRADE) assessment using the standard five GRADE criteria.^39^ We used ‘summary of findings’ tables to provide outcome-specific information related to the overall quality of evidence from studies included in comparisons, the magnitude of effect of the interventions examined and the sum of available data on the outcomes we considered.

### Data synthesis

Data regarding medication-related interventions were summarised and described separately using the TIDieR framework.^40^ Barriers and facilitators in the primary research papers were summarised to allow comparison with those already identified.^5^

Meta-analysis was planned if comparable studies (design and outcome(s) reported) were identified. Before meta-analysis, we transformed data onto the uniform log odds scale using the Comprehensive Meta-Analysis (V.3) software (Biostat, New Jersey, USA). Then for all of the comparable ‘uncontrolled before-and-after studies’ with the relevant outcome data we meta-analysed using the DerSimonian-Laired random-effects model.^41^ The Hartung-Knapp random-effects method for pooling^42^ was used instead of DerSimonian-Laird, as it is a more robust method of choice when study sizes are small and there is considerable heterogeneity, as likely to be present in the non-randomised uncontrolled trials identified.

Statistical heterogeneity for assessment of comparability of studies was undertaken by visual inspection of forest plots and I^2^ statistic (0%–25% low; ≥25%–74% substantial and ≥75% considerable heterogeneity) with the associated 95% CIs.^43^ For each meta-analysis with 10 studies or more, funnel plots, Begg’s and Egger’s test were used to examine potential publication bias. The trim-and-fill method was used as a sensitivity analysis to observe cases of small study publication bias.^44^ We planned subgroup analyses if sufficient data were available for studies with comparable interventions and outcome measures. All meta-analyses were performed in the statistical software R (V.4.0.3) with packages *meta* 45 and *metafor*.^46^

## Results

### Study inclusion

**Figure 1:**
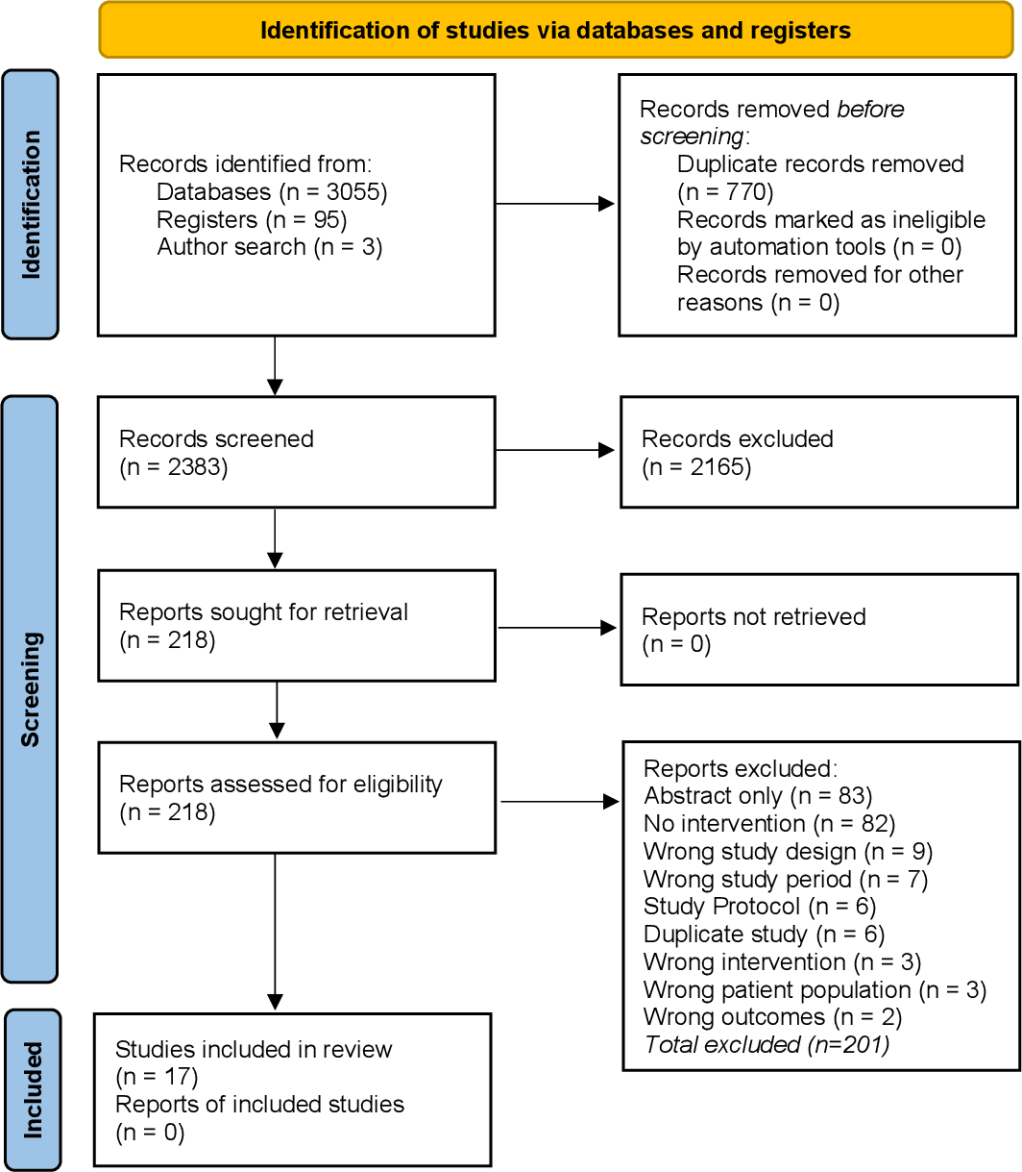
Preferred Reporting Items for Systematic Reviews and Meta-Analyses flow diagram.^30^

The literature searches identified 3153 references, after removal of duplicates, abstract and title screening and then assessment of full-text eligibility, 17 studies were included (table 1).^13 14 47–61^ The publications of Wohlt *et al* (before)^62^ and Hatch *et al* (after)^59^ were considered as a single study referenced as Hatch *et al*,^59^ for the purposes of this systematic review. The PRISMA flow chart for study inclusion is shown figure 1.

**Table 1:** Summary of study characteristics

Study/Country	Study design/centres (number)	Intervention target	Participants Inclusion criteria Exclusion criteria	Participant numbers (control/before: intervention/after)	Intervention description, (components) and (timing)
Anstey *et al* Australia^54^	Before-after (B-A); prospective; multi (n=5)	De-escalation of inappropriate stress ulcer prophylaxis (SUP)	Adult intensive care unit (ICU) patients (medical, surgical, cardiothoracic) Inclusion: consecutive ICU patients admitted during the active study period Exclusion: patients <18 years of age	842 (469: 373)	SUP de-escalation bundle (education of ICU medical staff; guidelines; pharmacist-led prescription discontinuation) (ICU stay)
Bosma *et al* Netherlands^13^	B-A; prospective; multi (n=2)	Medication errors (MEs) on ICU discharge	Adult ICU patients (medical, surgical, neurosurgery, cardiology) Inclusion: patients admitted on ≥1 regular medicine with ICU stay >24 hours. Discharge (disch) patients included if in admission (adm) study part, surviving ≥24 hours after ICU disch Exclusion: patients transferred to another hospital, adm and disch same weekend period, patients unable to understand Dutch or English	380 (203: 177)	Medicines reconciliation (med rec) at care transitions (by ICU pharmacist; in patient rounds; combined with medication review (med rev) by pharmacist with ICU medical staff review to create ICU disch medication list. Medication advice included as supplement to the ward discharge letter. Discharge medication prepopulated on the ward electronic (e-) prescribing system) (ICU adm, ICU stay, ICU disch)
Buckley *et al* USA^55^	B-A; retrospective; single	De-escalation of inappropriate SUP	Adult ICU patients (≥18 years) Inclusion: all patients receiving acid suppressing therapy (AST) Exclusion: patients on treatment for gastrointestinal (GI) disorders or admitted on AST. Patients in the emergency department, rehabilitation or psychiatric wards	341 (174: 167)	SUP de-escalation programme (pharmacist-led authorised stress ulceration prescription management) (ICU stay, ward stay)
Coon *et al* USA^56^	B-A; prospective; single	Med rec (of specific intravenous vasoactives)	Adult ICU patients (neurosciences) Inclusion: all consecutive ICU patients transferred to the ward	261 (130: 131)	Structured ICU handover checklist (incorporated into e-discharge documentation (by ICU medical staff)) (ICU disch)
D'Angelo *et al* USA^57^	B-A; retrospective; single	De-escalation of inappropriate antipsychotics	Adult ICU patients (medical) Inclusion: all ICU patients initiated on antipsychotic therapy for ICU delirium ≥24 hours prior to ward transfer	281 (140: 141)	Antipsychotic discontinuation bundle (education of medical, nursing and pharmacy staff; clinical guidelines (including non-pharmacological interventions and de-escalation based on delirium screening) (ICU stay)
Hammond *et al* USA^58^	B-A; retrospective; single	De-escalation of inappropriate SUP	Adult ICU patients (medical) ≥18 years Inclusion: all patients prescribed AST Exclusion: diagnosis of GI bleed, receiving AST on ICU adm, or history of Zöllinger-Ellison syndrome	219 (101: 118)	Educational interventions for SUP (education of ICU medical staff; guideline; pharmacist on ward rounds to support education) (ICU stay)
(B) Wohlt *et al* 62 (A) Hatch *et al* 59 USA	B-A; retrospective; single	De-escalation of inappropriate SUP	Adult ICU patients (medical, surgical) ≥18 years Inclusion: all ICU patients Exclusion: patients with a GI bleed, Zöllinger-Ellison syndrome, prisoner status or died in hospital	750 (394: 356)	Education on SUP (education of ICU and ward medical and pharmacy staff; audit and feedback of preintervention results; guideline) (ICU stay, ward stay)
Heselmans *et al* Belgium^14^	Randomised controlled trial; prospective; multi (n=3)	Drug-related problems in patients after ICU to ward transfer	ICU patients (medical, surgical) ≥15 years Inclusion: patients with ICU stay ≥3 days and transferred to surgical, medical or geriatric ward Exclusion: patients with a ‘do not resuscitate’ order	600 (299: 301)	Medication review by ward-based pharmacists after ICU patient transfer (ward stay (<48 hours of ICU transfer))
Kram *et al* USA^60^	B-A; retrospective; single	De-escalation of inappropriate antipsychotics	Adult ICU patients (medical, surgical, cardiothoracics, neurosciences and cardiac) ≥18 years Inclusion: ICU patients with atypical antipsychotic prescribed in ICU Exclusion: patients had <2 antipsychotic doses in ICU, on antipsychotics pre-ICU adm or non-delirium psychiatric indication, or if died in ICU	358 (133: 225)	E-handover tool (prompting medication review by pharmacists (ICU and ward); supported by education (pharmacy staff), including audit and feedback of preintervention results) (ICU stay; ICU disch; ward stay)
Medlock *et al* Netherlands^61^	B-A; prospective; single	ICU e-disch letter (template included med rec details)	Adult ICU patients (medical, surgical) Inclusion: all critical care patients (discharged alive or dead)	6823 (1872: 4951)	E-letter to ward medical staff and general practitioner (with template and automatic assignment to ICU medical staff) (ICU disch)
Meena *et al* USA^47^	B-A; retrospective; single	De-escalation of inappropriate SUP	Adult ICU patients Inclusion: all ICU patients Exclusion: patients already taking AST on admission, therapeutic indication for AST, patients died within 24 hours of admission	224 (106: 118)	Education sessions for medical staff (didactic education session for junior medical staff) (ICU stay)
Parsons Leigh *et al* Canada^48^	B-A; retrospective; single	ICU e-transfer tool with eight key elements (including active medicines and med rec)	Adult ICU patients (medical, surgical, neurosurgical and trauma) Inclusion: randomly selected cohort of ICU patients transferred to an inpatient ward Exclusion: ICU patients not transferred to an inpatient ward	60 (30: 30)	E-transfer tool (auto-population of elements, eg, medicines to continue on ward transfer with facility to review and refine; facility to compare with preadmission med rec and identify changes (by medical staff)) (ICU disch)
Pavlov *et al* USA^49^	B-A; retrospective; single	De-escalation of inappropriate SUP and bronchodilators	Adult ICU patients (medical, surgical) Inclusion: ICU patients on acid blockers or bronchodilators Exclusion: patients who died during their adm or still in ICU on study data extraction	454 (201: 253)	Med rec (on hospital adm (pharmacy technician) and ICU disch (ICU nurse), with medical staff confirmation and in reconciliation with medication on ICU disch) (ICU disch)
Pronovost *et al* USA^50^	Time-series analysis; prospective; single	MEs on ICU discharge	Adult ICU patients (surgical) Inclusion: random selection of 10–15 patients per week	No information	Med rec (by ICU nurses on patient adm and ICU disch. Specific MEs prompted discussion with ICU medical staff) (ICU adm, ICU disch)
Stuart *et al* USA^53^	B-A; retrospective; single	De-escalation of inappropriate antipsychotics	Adult ICU patients (medical, surgical, cardiac) Inclusion: ICU patients with antipsychotic prescribed for delirium Exclusion: palliative care or died, on antipsychotics pre-ICU adm, or non-delirium psychiatric indication	158 (79: 79)	Pharmacist-led de-escalation protocol (de-escalation guideline with education of staff (ICU and ward pharmacists). Pharmacists authorised to discontinue or taper antipsychotics in ICU patients with resolved delirium symptoms) (ICU stay (direct patient disch), ward stay)
Tasaka *et al* USA^51^	B-A; retrospective; single	De-escalation of inappropriate SUP	Adult ICU patients (medical, surgical) Inclusion: all ICU patients Exclusion: patients requiring continued AST (eg, active GI bleed), or no indication for AST (eg, total gastrectomy)	124 (74: 50)	SUP de-escalation bundle. Guideline, education of staff (medical, nurses, pharmacists, dietitians), multifaceted awareness campaign, pharmacist SUP recommendations (on care rounds, or by text/telephone) with documentation in e-medical notes. SUP not included in the e-prescribing core or ICU adm order sets (ICU stay)
Zeigler *et al* USA^52^	B-A; retrospective; single	De-escalation of inappropriate SUP	Adult ICU patients (medical, surgical) Inclusion: all ICU patients in study period Exclusion: patients receiving AST pre-ICU adm, or acute GI bleed indication, or if they died	114 (53: 61)	Med rec (by nurses and pharmacist with medical staff review. At care transitions and hospital disch. Training via classes, web-based module, hospital presentations and individual sessions. No SUP education given) (ICU adm, ICU disch, hospital disch)

### Characteristics of included studies

Most of the studies (76%) were from North America (n=12 the USA, n=1 Canada),^47–53 55–60^ three from Europe (n=2 The Netherlands, n=1 Belgium),^13 14 61^ and one from Australia^54^ (table 1). Only the study by Heselmans *et al* 14 was an RCT, 15 were uncontrolled before-after studies^13 47–49 51–61^ and one was an uncontrolled interrupted time-series analyses.^50^ Most studies (n=14, 82%) were conducted in single centres, three were multicentre studies.^13 14 54^ Approximately one-third of the studies (n=6, 35%) were prospective in data collection and evaluation.^13 14 50 54 56 61^ Interventions are fully described according to the TIDieR framework (online supplemental table S1).^40^

10.1136/bmjqs-2021-013760.supp2 Supplementary data

### Characteristics of interventions

#### Intervention components

Nine (53%) of the studies examined a single intervention component (table 1 and online supplemental table S1).^14 47–50 52 55 56 61^ The key intervention components described were education of staff, medication review, guidelines, electronic transfer/hand-over checklist or letter and medicines reconciliation. Multicomponent interventions,^51 53 54 57–59^ targeted inappropriate medication continuation at transfer points. These were based on education of staff and guidelines, with three studies also including a medication review element.^51 53 54^

##### Education of staff

Education of staff was an intervention component in eight studies (47%).^47 51 53 54 57–60^ Studies varied by healthcare professionals the education was targeted at. Uniprofessional,^47 53 54 58 60^ educational approaches were more commonly employed than multiprofessional.^51 57 59^ Mode of and frequency of delivery varied across the studies (table 1 and online supplemental table S1).

##### Medication review

Seven studies included a specific medication review component within the intervention^13 14 51 53–55 60^ (table 1 and online supplemental table S1). Five of these studies included a pharmacist-led medication review as part of a multicomponent intervention targeted at a specific de-prescribing initiative, either of inappropriate SUP,^51 54 55^ or use of antipsychotics.^53 60^

##### Guidelines

Six studies (35%) implemented a clinical guideline intervention component,^51 53 54 57–59^ four focused on de-escalation of inappropriate medication on transfer^51 53 54 57^ (table 1 and online supplemental table S1). Three studies had clear multiprofessional participation in the guideline development.^51 57 58^ Implementation of the clinical guidelines included education and awareness of staff in all studies.^51 53 54 57–59^

##### Medicines reconciliation

Four studies investigated a medicines reconciliation intervention (table 1 and online supplemental table S1).^13 49 50 52^ The healthcare professionals undertaking the medication review varied, with pharmacist,^13^ pharmacy technician,^49^ ICU nurse^50^ and ICU nurse with a pharmacist involved.^52^ All medicines reconciliation processes required review and authorisation by medical staff, with pharmacist medication review and advice also provided in the study by Bosma *et al*.^13^ Medicines reconciliation was undertaken on patient ICU admission and ICU discharge in all four studies.^13 49 50 52^ Bosma *et al* 13 provided detailed information on the delivery and quality of medicines reconciliation on the interfaces of care.

##### Electronic transfer/handover tool or letter

Four studies^48 56 60 61^ implemented an intervention designed to improve communication from the ICU to the hospital ward and included a medication-related component (table 1 and online supplemental table S1). The communication was directed at a range of healthcare professionals: general practitioners,^61^ ICU and hospital ward pharmacists^60^ and hospital ward medical staff.^48 56^

#### Intervention timing

There was a high degree of variation with regard to the timing of the intervention components delivered in the patient acute care pathway (table 1 and online supplemental table S1). Most (n=10, 59%) of studies investigated an intervention delivered at a single time-point in the patient pathway.^14 47–49 51 54 56–58 61^ For five of these studies, this was during the ICU patient stay,^47 51 54 57 58^ which was also the period most interventions included (n=10, 59%).^13 47 51 53–55 57–60^ Eight studies (47%) also included the ICU discharge period in the intervention delivery.^13 48–50 52 56 60 61^

Five studies included an intervention element that included the patient’s hospital ward stay,^14 53 55 59 60^ three of which included a medication review interventional component.^14 55 60^

#### Facilitators and barriers to intervention delivery

Most studies (12, 71%) identified facilitators to the intervention delivery (table 2).^13 48 50 51 53–56 58–61^ The availability of specialist ICU clinical pharmacists contributing to a range of activities including multiprofessional ward rounds, staff education, medicines reconciliation, medication reviews and de-prescribing were highlighted in seven studies.^13 51 53–55 58 59^ Integration of the electronic transfer/handover tool or letter into existing electronic systems was reported as a facilitator in three studies,^48 56 61^ as was auto-population of data on transfer reports,^48 50 61^ and software tailoring.^48 60 61^ The important role of a supportive quality improvement organisational culture was emphasised in two studies.^50 61^

**Table 2:** Facilitators and barriers identified from the selected studies classified by system factors^77^

System factor	Facilitator/Barrier	Studies, n (%)
Healthcare professionals		
Clinical pharmacist availability	Facilitator	7 (41%)^13 51 53–55 58 59^
Multiprofessional collaboration	Both (facilitator when good collaboration, barrier when poor collaboration)	3 (18%)^14 50 51^
Staff perception of limited intervention value	Barrier	2 (12%)^52 56^
Off shift hours (eg, clinical pharmacists)	Barrier	2 (12%)^13 53^
Tasks		
Pharmacist participation on ICU multiprofessional ward round	Facilitator	4 (24%)^13 51 58 59^
Increased workload associated with discharge intervention process (eg, medicines reconciliation, checklist)	Barrier	3 (18%)^13 50 56^
Structured approach to medicines reconciliation	Facilitator	2 (12%)^13 50^
Gaps in educational process	Barrier	2 (12%)^52 58^
Education package revised, condensed and delivered regularly	Facilitator	1 (6%)^51^
Focus on the care transition	Facilitator	1 (6%)^13^
Technologies and tools		
Auto-population of discharge information from electronic health record	Facilitator	3 (18%)^48 50 61^
Checklist integrated into existing work flow/systems	Facilitator	3 (18%)^48 56 61^
Tailored discharge letter/tool software	Facilitator	3 (18%)^48 60 61^
Guideline and supporting documentation	Facilitator	1 (6%)^59^
Organisational conditions		
Quality improvement culture	Facilitator	2 (12%)^50 61^
Task allocation	Both	2 (12%)^50 61^
Ability to initiate the summary on patient admission and edit throughout the ICU stay	Facilitator	1 (6%)^48^
Patient discharged from ICU out of hours	Barrier	1 (6%)^13^
Short discharge time-frame	Barrier	1 (6%)^13^

ICU, intensive care unit.

Fewer studies (n=7, 41%) provided an indication of intervention barriers (table 2).^13 14 50 52 53 56 58^ The most common barrier cited (n=3, 18%) was increased workload associated with the intervention.^13 50 56^ Multiprofessional collaboration was reported as barrier to intervention delivery when limited,^14^ and a facilitator when effective.^50 51^

### Outcomes

#### Medication outcomes

We describe the medication outcomes according to the focus of the intervention on those outcomes.

There was sufficient de-prescribing medication outcome data from intervention studies to conduct a meta-analysis. A narrative synthesis of other medication outcome data and findings related to interventions with medicines optimisation, patient and economic evaluation outcomes are presented. Most studies used medical notes review for medication outcomes. A summary of the study outcomes and measures is shown online supplemental table S2.

10.1136/bmjqs-2021-013760.supp3 Supplementary data

##### De-prescribing outcomes

Eleven studies (65%) focused on de-escalation of inappropriate medication therapy on ICU discharge or at hospital discharge (table 1),^47 49 51–55 57–60^ the most common focus being the reduction of inappropriate SUP.^47 51 52 54 55 58 59^

##### Medicines optimisation outcomes

Interventions targeted on medicines optimisation outcomes had a much broader remit, examining the clinical effectiveness and safety of all the patient’s medicines, usually employing a combination of medicines reconciliation and medication review.^63^ Heselmans *et al* 14 focused on a medication review intervention conducted in three Belgian hospitals by hospital pharmacists on the ward within 48 hours of the ICU patient transfer; 54.1% (203/375) of the drug-related problems (DRPs) were adjusted on time in the intervention group compared with 12.8% (47/368) in the control group. Compared with the control group, the odds of implementing a change in medication therapy recommendations in the intervention group were 10-fold higher (OR 10.1, 95% CI 6.3 to 16.1), increasing to OR 15.6 (95% CI 9.4 to 25.9), when between-group differences in types of DRPs were accounted for.

In a two-centre before-after study, Bosma *et al* 13 investigated the effect of a medicines reconciliation intervention on discharge medication transfer errors (any unintentional discrepancy between the patient’s prescription chart and best possible ward medication list at 24 hours after discharge) (online supplemental table S2). On ICU discharge, 41.2% (73/177) of patients in the intervention period had at least one ME compared with 73.9% (150/203) of patients in the before phase. After correcting for baseline differences in patient severity of illness, patients in the intervention period had an adjusted OR 0.24 (95% CI 0.15 to 0.37) for a discharge ME.

##### Documentation and communication outcomes

Electronic transfer/handover tool or letters improved the timeliness of information transfer and completeness of information provision.^48 61^

#### Patient outcomes

Eight studies (47%) reported between-group comparison of patient outcomes (online supplemental table S2).^13 14 49 53 56 58 60 61^ No mortality difference was reported in the medication review RCT,^14^ or in one communication before-after intervention study.^61^ Two studies included an assessment of actual,^56^ or potential ADEs,^13^ with a significant reduction in the latter.^13^ The RCT investigating medication reviews, reported no differences between the intervention and control groups in any patient outcomes (mortality rate, ICU readmission rate or hospital length of stay).^14^ Three other studies reported no effect of the intervention on hospital length of stay.^53 56 60^

##### Risk of bias and overall quality of evidence

Overall, the quality of the studies was low (online supplemental figures S1 and S2). All the non-randomised studies were assessed as moderate to serious RoB except one,^50^ which was graded as critical RoB and was excluded from the meta-analysis. The RCT by Heselmans *et al* 14 was also assessed as high RoB due to the randomisation process domain.

10.1136/bmjqs-2021-013760.supp4 Supplementary data

The GRADE assessments for the overall quality of evidence for the main meta-analysis and subgroup analysis of studies focused on the de-escalation of inappropriate medication on ICU and hospital discharge are shown (online supplemental table S3).

#### Main meta-analysis

**Figure 2:**
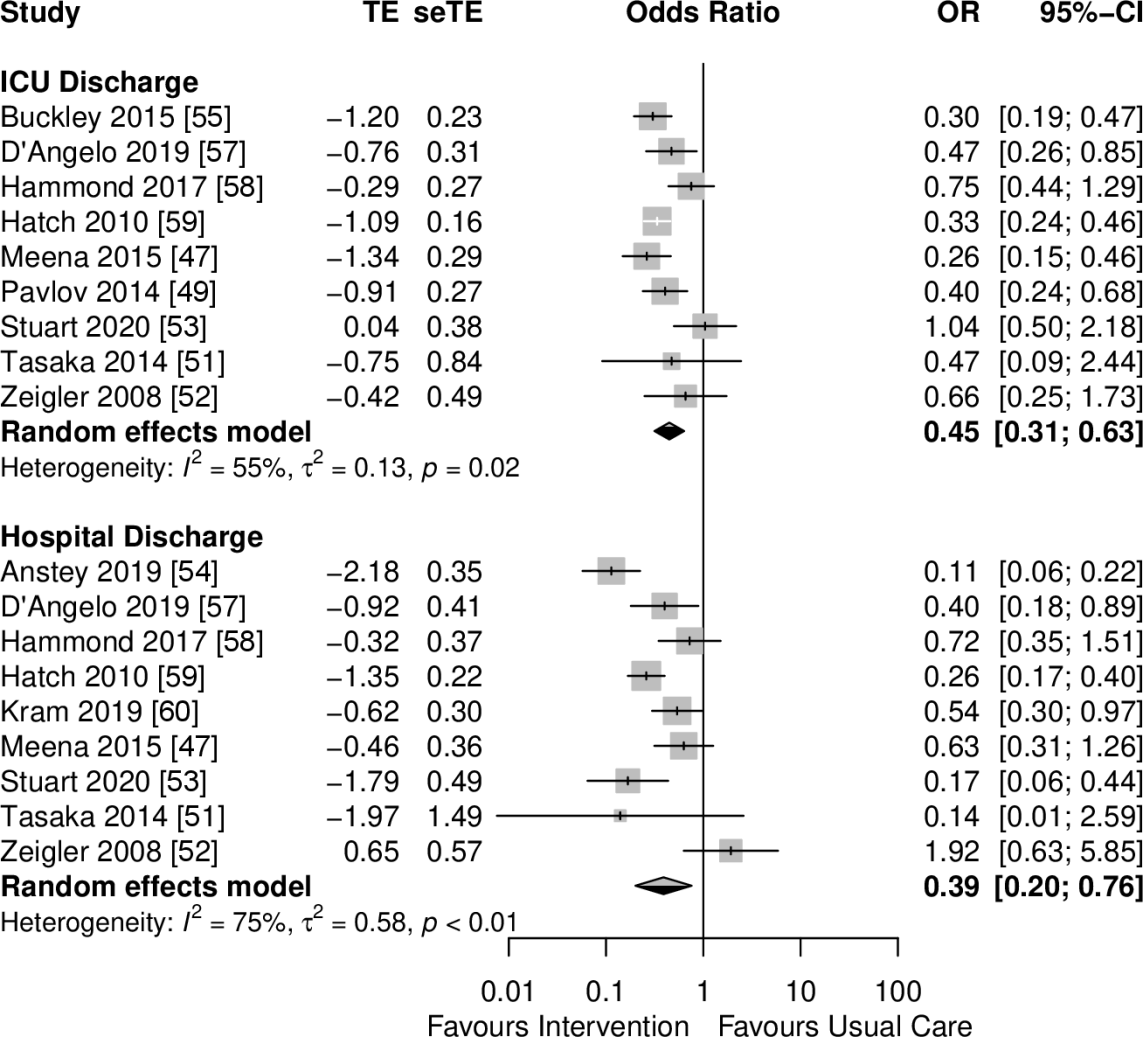
Main meta-analysis. TE: log OR; seTE: SE of log OR.

The meta-analysis was undertaken for interventions that had the common outcome of de-escalation of inappropriate medication (de-prescribing) at ICU and hospital discharge points (figure 2). Compared with the before period of usual care, pooled analysis of all interventions reduced the risk of inappropriate medication continuation at ICU discharge (OR=0.45 (95% CI 0.31 to 0.63), I^2^=55 (5–79) %, n=9 studies) and hospital discharge (OR=0.39 (95% CI 0.2 to 0.76), I^2^=75 (52–87) %, n=9 studies). There was no evidence of publication bias as indicated by funnel plot symmetry, and the non-significant Egger’s regression test (p=0.583) and trim-and-fill method (p=0.152) for the primary outcome (online supplemental figure S3). The quality of evidence for ICU discharge and hospital discharge points were considered low to very low, respectively (online supplemental table S3).

**Figure 3:**
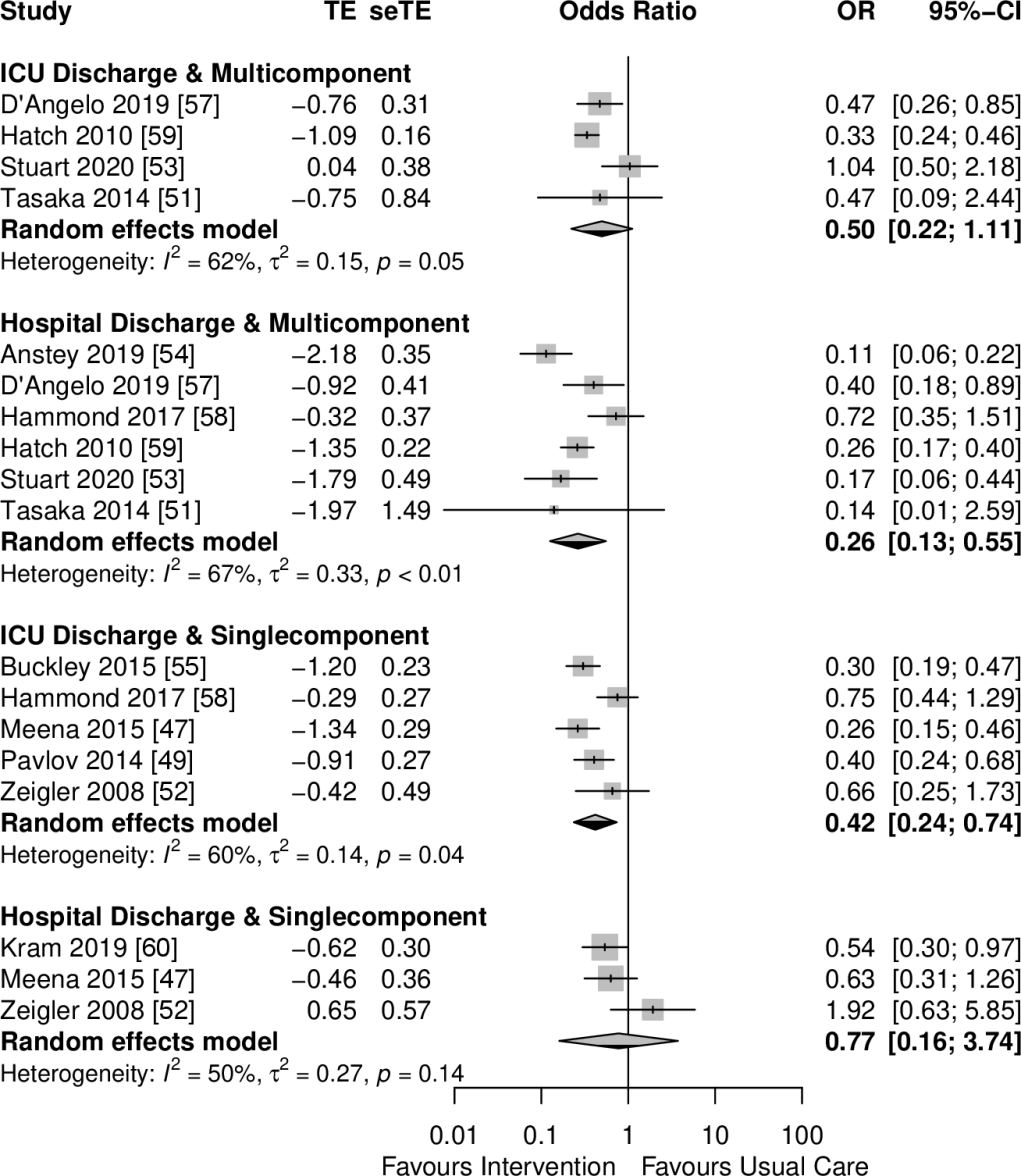
Subgroup meta-analysis. TE: log OR; seTE: SE of log OR.

#### Subgroup analysis

Subgroup meta-analysis showed that multicomponent interventions (based on education of staff and guidelines) led to a reduction in inappropriate medication continuation at the hospital discharge point (OR=0.26 (95% CI 0.13 to 0.55), I^2^=67 (21–86) %, n=6 studies). There was an indication of benefit at ICU discharge, but this did not reach statistical significance (OR=0.5 (95% CI 0.22 to 1.11), I^2^=62 (0–87) %, n=4 studies) (figure 3). Quality of evidence was moderate at hospital discharge and low for the ICU transition point (online supplemental table S3). In contrast, single component interventions (education of staff, medicines reconciliation, medication review) demonstrated a reduction in inappropriate medication continuation at ICU discharge (OR=0.42 (95% CI 0.24 to 0.74), I^2^=60 (0–85) %, n=5 studies), but not hospital discharge (OR=0.77 (95% CI 0.16 to 3.74), I^2^=50 (0–86) %, n=3 studies) (education of staff, medicines reconciliation, electronic handover tool) (figure 3); quality of evidence was graded low and very low, respectively (online supplemental table S3). No further subgroup analyses of medication review and medicines reconciliation interventions were undertaken given the small number of studies involving high levels of heterogeneity and imprecise data.

## Discussion

### Summary of main findings

This systematic review has found that pooled analysis of specific interventions (education of staff, medication review, guidelines, medicines reconciliation, electronic handover tool) to reduce inappropriate medication continuation on ICU and hospital discharge were effective, with more than twice the likelihood of effective de-prescribing (low and very low quality of evidence). Multicomponent interventions based on education of staff and guidelines were associated with an almost fourfold higher rate of inappropriate medication discontinuation at hospital discharge compared with usual care, with moderate quality of evidence. Single component interventions (education of staff, medicines reconciliation, medication review) were twice as likely as usual care to reduce inappropriate medication continuation at ICU discharge only (shown by low quality of evidence).

Patient and economic evaluation outcomes were reported in a minority of studies. Only one study demonstrated a reduction in potential ADEs with an ICU medicines reconciliation intervention,^13^ also providing the most compelling health economic data. Structured electronic transfer/handover tool or letters both within ICU, and beyond ICU to the hospital ward and general practitioners, improved medication-related completeness and communication.

Successful delivery of complex interventions usually requires a combination of several factors including resources, education and training of staff.^64^ Medication review by hospital ward-based clinical pharmacists soon after ICU patient transfer was very effective in the reduction of clinically important DRPs.^14^ Furthermore, medicines reconciliation by clinical pharmacists prior to ICU patient transfer to the hospital ward also effectively reduced MEs and potential ADEs.^13^ The effectiveness of implementing medicines reconciliation interventions can vary,^65^ being prone to limitations in healthcare staff collaboration and task allocation,^66^ that may contribute to the lack of an association between ICU patient outcomes and medicines reconciliation services as reported by van Sluisveld *et al*.^24^ Moreover, the ability to provide discharge medicines reconciliation 7 days per week, at a time-pressurised point of care, is an important barrier to delivery.^13 50^ It is unknown how such variation in system resources and intervention delivery affect the risks of adverse patient outcomes with out-of-hours ICU to hospital ward transfers.^67^ However, in a retrospective evaluation of adherence to medicines optimisation in ICU survivors of sepsis, delivery of medicines optimisation was associated with improved patient mortality at 90 days.^27^

Despite engagement of patients and family being an identified a facilitator to the provision of high-quality care for ICU patients transferring to a hospital ward,^5 68^ and highlighted in the WHO technical report,^4^ none of the medication-related intervention studies included any patient or family considerations. Given the complexity of the medication changes ICU patients experience, patients and family are an important contributor in the understanding and continuity of medication in transitions of care, for example, self-management.^69^ Nevertheless, indications from ICU follow-up clinics^19^ suggest an ICU culture shift is needed to develop a partnership between healthcare professionals, patients and families for this medicines optimisation aspect.^70^

### Comparison with previous research

The main meta-analysis results are consistent with a recent systematic review of pharmacy-supported interventions to reduce inappropriate continuation of SUP in patients after ICU and hospital discharge that reported similar effectiveness.^29^ However, our meta-analyses considered discontinuation of all potentially inappropriate medication and were unconstrained by interventions delivered by a single profession. Multicomponent de-prescribing interventions primarily consisted of policy-type (guidelines) and single intervention functions (education (and training) of staff), sometimes supported by audit and feedback, to affect staff behaviour change and likely intervention adoption.^71^ This combination re-enforces information and desired practice, which have been reported to be effective in changing healthcare staff behaviours in de-prescribing interventions.^72^ A recent systemic review of medication-related interventions delivered in hospital and following discharge^71^ reported that the size of the treatment effect increased with the intensity (factoring number and repetition of components) of medication-related intervention delivered. However, such intervention intensity needs to be balanced with routine deliverability.

The identified facilitators and barriers to medication-related interventions are consistent with, and add further specific detail to, those already identified in other wider reviews of interventions to improve ICU patient continuity of care.^5 68 73^ These facilitators and barriers should inform the development and implementation of medication outcome interventions, particularly when staff behaviour change across different professions and teams is required.^74^

### Strengths and limitations

This systematic review had several strengths including multidisciplinary team expertise, comprehensive searches, detailed critical appraisal of the studies and presentation of different study elements. However, there are also limitations. The conclusions are limited by the availability of study designs (uncontrolled before-after) employed, with inherent RoB that was mainly due to baseline confounding risks. We did not have sufficient studies to undertake a sensitivity analysis to examine the impact of the RoB assessment, although since the vast majority of studies were high risk, a sensitivity analysis is very unlikely to show any different findings. The meta-analysis was limited to a subgroup outcome of medicines optimisation, that is, de-prescribing of SUP, antipsychotics and bronchodilators. Wider de-prescribing foci are required, for example, opioid analgesia, to address the risk of inappropriate long-term continuation.^19^ Although studies used similar definitions for the respective outcomes of multiple inappropriate medications, as well as use of patient chart reviews, there are still significant inconsistencies in terms of design and exact methods. We were unable to describe the intervention mechanisms of action as most studies neglected to provide process evaluation elements.

### Implications for policy and practice

Intervention to reduce medication-related harm for patients on transitions of care is a policy and practice priority,^3^ being particularly pertinent to the complexity of acutely ill patient care transition to a hospital ward.^75^ Our findings suggest that multicomponent interventions, including education of staff and guidelines, are promising in reducing inappropriate continuation of acute medication by hospital discharge (moderate quality of evidence). It seems reasonable to consider routine adoption of these low-risk and likely low-cost interventions. However, further research is required on how best to improve the more challenging medicines optimisation aspect, re-introduction of clinically important chronic medication, for ICU patients transferring to the ward and beyond. To inform this research, consensus on the key medication-related interventions to use and what medication and patient outcomes measures to test these against, as well as how the human factors involved in medication transfer errors can be addressed are required. The resulting intervention is likely to be a theory-informed multicomponent intervention that should be evaluated in a multicentre cluster or stepped-wedge RCT that includes a health economic evaluation. Process evaluation capable of informing the complex intervention mechanism of action and theory is also needed. In turn, this would support the implementation and delivery of practice and future policy recommendations.^76^

## Conclusions

This systematic review and meta-analysis newly identified that interventions aimed at reducing inappropriate medication continuation on patient ICU discharge and hospital discharge increased de-prescribing efficacy. Multicomponent interventions, built on education of staff and guidelines, appeared most effective in reducing inappropriate medication by hospital discharge. However, none of the de-prescribing initiatives exhibited beneficial effects on patient outcomes. More complex interventions such as medication review and medicines reconciliation, targeted at reducing MEs and medication-related problems on ICU discharge, were very effective and reduced potential ADEs. Our findings highlight the need to improve the quality and design of future prospective randomised intervention studies in this area.

## Data Availability

All data relevant to the study are included in the article or uploaded as supplementary information.
