# Supplementary material for: Medication-related interventions to improve medication safety and patient outcomes on transition from adult intensive care settings: a systematic review and meta-analysis
Source: BMJ Qual Saf. 2022 Jan 18;31(8):609–22. doi: 10.1136/bmjqs-2021-013760 (PMC9304084; doi:10.1136/bmjqs-2021-013760)
Supplement: Supplementary data [file bmjqs-2021-013760supp001.pdf]

Additional File 1. Search strategy

A systematic search strategy was developed on MEDLINE by the information specialist (*EEE*) in consultation with the lead reviewer (*AAA*), to identify literature on medication-related interventions in the population of intensive care patients on transition to a hospital ward. The pilot search strategy was run on MEDLINE and the results shared with the lead reviewer to check that known studies were retrieved by the search. One known study was not retrieved, so additional search terms were added to improve the sensitivity of the search. The final search strategy was circulated to the review team for comment and no further changes were required.

The search strategy used a combination of free-text and thesaurus searching (where available). All databases were searched from inception to present, and no search limits were applied. The searches were run in October 2020 on the following sources:

- Ovid MEDLINE(R) and Epub Ahead of Print, In-Process & Other Non-Indexed Citations and Daily 1946 to October 09, 2020
- International Pharmaceutical Abstracts (via Ovid)
- Embase 1974 to 2020 October 13
- CINAHL via EBSCO (1981-present)
- Cochrane Database of Systematic Reviews Issue 10 of 12, October 2020
- Cochrane Central Register of Controlled Trials Issue 10 of 12, October 2020
- Science Citation Index Expanded (SCI-EXPANDED) --1900-present (via Web of Science)

Complete search strategies are provided below.

**Database**	**Date Searched**	**Number of References Retrieved (including duplicates)**
Ovid MEDLINE(R) and Epub Ahead of Print, In-Process & Other Non-Indexed Citations and Daily 1946 to October 09, 2020	12/10/2020	546
International Pharmaceutical Abstracts (via Ovid)	13/10/2020	189
Embase 1974 to 2020 October 13	14/10/2020	807
CINAHL via EBSCO	15/10/20	343
Cochrane Database of Systematic Reviews Issue 10 of 12, October 2020	15/10/20	60

Cochrane Central Register of Controlled Trials<br>Issue 10 of 12, October 2020	15/10/20	501
Science Citation Index Expanded (SCI-EXPANDED) --1900-present (via Web of Science)	16/10/20	609
**Total number of references retrieved (including duplicates)**		**3,055**

Search Strategies

MEDLINE

Database: Ovid MEDLINE(R) and Epub Ahead of Print, In-Process & Other Non-Indexed Citations and Daily <1946 to October 09, 2020>

Search Strategy:

----

1 exp PATIENT DISCHARGE/ (30323)

2 exp Patient Transfer/ (8558)

3 (discharg* or transition* or transfer*).ti,ab. (1361480)

4 exp "Continuity of Patient Care"/ (247491)

5 or/1-4 (1566670)

6 exp MEDICATION RECONCILIATION/ (1167)

7 exp Inappropriate Prescribing/ (3395)

8 exp Medication Errors/ (17485)

9 exp Medication Systems/ (5510)

10 exp Medication Therapy Management/ (2192)

11 exp Drug Utilization/ (25613)

12 exp Potentially Inappropriate Medication List/ (507)

13 exp Pharmaceutical Preparations/ and (exp "Process Assessment (Health Care)"/ or exp "Continuity of Patient Care"/) (1474)

14 ((medication* or medicine* or drug* or prescribing or prescription*) adj1 (review* or reconciliation or optimi?ation or system* or error* or discrepanc* or safety)).ti,ab. (26424)

15 ((medication* or medicine* or drug* prescribing or prescription*) and (adverse adj1 event*)).ti,ab. (22086)

16 ((medication* or medicine* or drug* or prescribing or prescription*) adj3 intervention*).ti,ab. (13076)

17 (communication adj5 (tool* or intervention* or system*)).ti,ab. (20641)

18 or/6-17 (120182)

19 5 and 18 (10151)

20 exp Critical Care/ (58522)

21 exp Intensive Care Units/ (85997)

22 exp Critical Illness/ (29697)

23 (ITU or ICU or ICUs or intensive care or critical care).ti,ab. (200296)

24 or/20-23 (258059)

25 19 and 24 (546)

****

IPA

Ovid Technologies, Inc. Email Service

Undertaken 13th October 2020
Search for: 1 and 4 and 10

Results: 189

Database: International Pharmaceutical Abstracts <1970 to September 2020>
Search Strategy:
----
1 (Intensive care or critical care or critical care unit$ or intensive care unit$ or critically ill or critical illness or ITU$ or ICU$).af. (8706)
2 (PATIENT DISCHARGE or Patient Transfer or Continuity of Patient Care or patient transition).af. (184)
3 (discharg$ or transition$ or transfer$).af. (21460)
4 2 or 3 (21480)
5 (MEDICATION RECONCILIATION or Medicines reconciliation or Medication Errors or Inappropriate Prescribing or Medication Systems or Drug Utilization or Medication Review or

Potentially Inappropriate Medication).af. (19805)
6 ((medication$ or medicine$ or drug$ or prescribing or prescription$) adj1 (review$ or reconciliation or optimi?ation or system$ or error$ or discrepanc$ or safety)).af. (18750)
7 ((medication$ or medicine$ or drug$ prescribing or prescription$) and (adverse adj1 event$)).af. (3694)
8 ((medication$ or medicine$ or drug$ or prescribing or prescription$) adj3 intervention$).af. (10351)
9 (communication adj5 (tool$ or intervention$ or system$)).af. (1266)
10 5 or 6 or 7 or 8 or 9 (44262)
11 1 and 4 and 10 (189)

Embase

Database: Embase <1974 to 2020 October 13>
Search Strategy:
----
1 *hospital discharge/ (13169)
2 *patient transport/ (8305)
3 (discharg* or transition* or transfer*).ti,ab. (1583038)
4 *patient care/ (68899)
5 or/1-4 (1652960)
6 *medication therapy management/ (4369)
7 *inappropriate prescribing/ (1551)
8 *medication error/ (8374)
9 *hospital organization/ (6476)
10 *drug utilization/ (5938)
11 *potentially inappropriate medication/ (917)
12 *drug/ (24905)
13 *health care quality/ (73500)
14 4 or 13 (139835)
15 12 and 14 (149)
16 ((medication* or medicine* or drug* or prescribing or prescription*) adj1 (review* or reconciliation or optimi?ation or system* or error* or discrepanc* or safety)).ti,ab. (42305)
17 ((medication* or medicine* or drug* prescribing or prescription*) and (adverse adj1 event*)).ti,ab. (38470)
18 ((medication* or medicine* or drug* or prescribing or prescription*) adj3 intervention*).ti,ab. (19390)
19 (communication adj5 (tool* or intervention* or system*)).ti,ab. (24704)
20 or/6-11 (26781)
21 or/15-20 (140206)
22 *intensive care/ (62895)
23 *intensive care unit/ (34671)
24 *critical illness/ (12110)
25 (ITU or ICU or ICUs or intensive care or critical care).ti,ab. (305297)
26 or/22-25 (333889)
27 5 and 21 and 26 (907)
28 limit 27 to (conference abstracts or embase) (807)

****

CINAHL

S28	S22 AND S27
S27	S23 OR S24 OR S25 OR S26
S26	TI (ITU or ICU or ICUs or intensive care or critical care) OR AB (ITU or ICU or ICUs or intensive care or critical care)
S25	(MH "Critical Illness")
S24	(MH "Intensive Care Units+")
S23	(MH "Critical Care+")
S22	S5 AND S21
S21	S6 OR S7 OR S8 OR S9 OR S10 OR S11 OR S16 OR S17 OR S18 OR S19 OR S20
S20	TI (communication N5 (tool* or intervention* or system*)) OR AB (communication N5 (tool* or intervention* or system*))
S19	TI ((medication* or medicine* or drug* or prescribing or prescription*) N3 intervention*) OR AB ((medication* or medicine* or drug* or prescribing or prescription*) N3 intervention*)
S18	TI ((medication* or medicine* or drug* prescribing or prescription*) and (adverse N1 event*)) OR AB ((medication* or medicine* or drug* prescribing or prescription*) and (adverse N1 event*))

S17	TI ((medication* or medicine* or drug* or prescribing or prescription*) N1 (review* or reconciliation or optimi?ation or system* or error* or discrepanc* or safety)) OR AB ((medication* or medicine* or drug* or prescribing or prescription*) N1 (review* or reconciliation or optimi?ation or system* or error* or discrepanc* or safety))
S16	S12 AND S15
S15	S13 OR S14
S14	(MH "Continuity of Patient Care+")
S13	(MH "Process Assessment (Health Care)+")
S12	(MH "Drugs+")
S11	(MH "Drug Utilization+")
S10	(MH "Medication Management")
S9	(MH "Medication Systems")
S8	(MH "Medication Errors+")
S7	(MH "Inappropriate Prescribing")
S6	(MH "Medication Reconciliation")

S5	S1 OR S2 OR S3 OR S4
S4	(MH "Continuity of Patient Care+")
S3	TI ((discharg* or transition* or transfer*)) OR AB ((discharg* or transition* or transfer*))
S2	(MH "Transfer, Discharge")
S1	(MH "Patient Discharge")

Cochrane Library

#1 MeSH descriptor: [Patient Discharge] explode all trees
#2 MeSH descriptor: [Patient Transfer] explode all trees
#3 ((discharg* or transition* or transfer*)):ti,ab,kw (Word variations have been searched)
#4 MeSH descriptor: [Continuity of Patient Care] explode all trees
#5 #1 or #2 or #3 or #4
#6 MeSH descriptor: [Medication Reconciliation] explode all trees
#7 MeSH descriptor: [Inappropriate Prescribing] explode all trees
#8 MeSH descriptor: [Medication Errors] explode all trees
#9 MeSH descriptor: [Medication Systems] explode all trees
#10 MeSH descriptor: [Medication Therapy Management] explode all trees
#11 MeSH descriptor: [Drug Utilization] explode all trees
#12 MeSH descriptor: [Potentially Inappropriate Medication List] explode all trees
#13 MeSH descriptor: [Pharmaceutical Preparations] explode all trees
#14 MeSH descriptor: [Process Assessment, Health Care] explode all trees
#15 MeSH descriptor: [Continuity of Patient Care] explode all trees
#16 #14 or #15
#17 #13 and #16
#18 (((medication* or medicine* or drug* or prescribing or prescription*) NEAR/1 (review* or reconciliation or optimi?ation or system* or error* or discrepanc* or safety))):ti,ab,kw (Word variations have been searched)
#19 (((medication* or medicine* or drug* prescribing or prescription*) and (adverse NEAR/1 event*))):ti,ab,kw (Word variations have been searched)
#20 (((medication* or medicine* or drug* or prescribing or prescription*) NEAR/3 intervention*)):ti,ab,kw (Word variations have been searched)
#21 ((communication NEAR/5 (tool* or intervention* or system*))):ti,ab,kw (Word variations have been searched)
#22 {OR #6-#12, #17, #18-#21}
#23 #5 and #22

#24 MeSH descriptor: [Critical Care] explode all trees
#25 MeSH descriptor: [Intensive Care Units] explode all trees
#26 MeSH descriptor: [Critical Illness] explode all trees
#27 ((ITU or ICU or ICUs or intensive care or critical care)):ti,ab,kw (Word variations have been searched)
#28 #24 or #25 or #26 or #27
#29 #23 and #28

Science Citation Index

#1 TOPIC: ((discharg* or transition* or transfer*))
#2 TOPIC: (((medication* or medicine* or drug* or prescribing or prescription*) NEAR/1 (review* or reconciliation or optimi?ation or system* or error* or discrepanc* or safety)))
#3 TOPIC: (((medication* or medicine* or drug* prescribing or prescription*) and (adverse NEAR/1 event*)))
#4 TOPIC: (((medication* or medicine* or drug* or prescribing or prescription*) NEAR/3 intervention*))
#5 TOPIC: ((communication NEAR/5 (tool* or intervention* or system*)))
#6 #5 OR #4 OR #3 OR #2
#7 TOPIC: ((ITU or ICU or ICUs or intensive care or critical care))
#8 #7 AND #6 AND #1

Grey Literature Search

A search of the trial registries International Clinical Trials Registry Platform (ICTRP) and ClinicalTrials.gov was conducted on 4th-5th August 2021. A series of targeted searches for intervention terms such as “medication review” “reconciliation” or “optimization” and setting terms such as “intensive care” or “critical care” were conducted and results retrieved were screened for relevance by the Information Specialist (*EEE*). 95 trials were found for inclusion consideration.
