# Supplementary material for: Medication-related interventions to improve medication safety and patient outcomes on transition from adult intensive care settings: a systematic review and meta-analysis
Source: BMJ Qual Saf. 2022 Jan 18;31(8):609–22. doi: 10.1136/bmjqs-2021-013760 (PMC9304084; doi:10.1136/bmjqs-2021-013760)
Supplement: Supplementary data [file bmjqs-2021-013760supp002.pdf]

Additional File 2: Intervention Study Template for Intervention Description and Replication (TIDieR) Summary

**Author/ Year**	**Brief Name**	**Why**	**What**	**Who Provided**	**How**	**Where**	**When and How much**	**Tailoring**	**Modifications**	**How well**
**Anstey 2019** [54]	Stress Ulceration Prophylaxis (SUP) de-escalation bundle	To improve SUP prescription compliance (including de-prescribing). To reduce medication costs in patients admitted to the intensive care units (ICU)	1. Site-based dissemination and education of locally produced SUP prescribing guidelines for medical staff (including documentation of indication and duration of therapy). 2. ICU pharmacist-led discontinuation of SUP prior to ICU discharge if no clear ongoing indication	ICU pharmacists	All sites had pharmacists present in the ICU in both study periods and used paper-based records throughout for SUP prescription review	ICUs (multicentre)	No information	No information	No information	No information
**Bosma 2018** [13]	Medicines reconciliation on ICU admission and discharge	Many changes are made to a patient's medication whilst in ICU. When an ICU patient is ready for ward	1. Creation of an accurate medication history list on ICU admission. 2. Creation of an ICU medication	ICU pharmacist created the medicines reconciliation lists. ICU discharge medicines	Medicines reconciliation on admission included contact with community pharmacy	ICUs (two centres). ICU pharmacists required for admissions, ward rounds and	Medicines reconciliation conducted on admission, ICU WRs and ICU patient	No information	No information	No information on how many patients were excluded (e.g.,

		transfer, there is an increased risk of medication errors as a result of failure to restart important chronic medication and/or potentially inappropriate medication is continued. Medicines reconciliation on ICU admission and discharge can help identify medication changes, medication transfer errors and reduce potential adverse drug events	discharge list sent as a section of the ICU discharge letter to the ward physician. 3. ICU pharmacist used medication history to inform advice during ICU ward rounds. 4. ICU medication review, advice and discussion with ICU physician regarding ward medication continuity plan. 5. Ward medication was pre-populated by the ICU pharmacist on the ward e-prescribing system	reconciliation done in conjunction with ICU and then ward medical staff	and hospital databases information (not stipulated if electronic or telephone) and face to face discussion with patient/relative. Medicines reconciliation then followed up with face-to-face discussion of ICU pharmacist recommendations with ICU medical staff. On ICU discharge the ICU pharmacist and ICU physician discussed the list and pharmacist recommendations.	planning on ICU to ward electronic prescription	discharge. Admission medicines reconciliation 87.3% (185) patients on admission and 68.9% (122) of patients on ICU discharge. Medicines reconciliation on ICU admission took a mean 24.0 (34.3) minutes; on ICU discharge it took a mean 29.4 (42.0) minutes			transfer to another hospital, both admission and discharge within the same weekend period and patient's inability to be counselled in Dutch or English). Quality of medicines reconciliation on ICU admission: Optimal 129 (60.8%); no (proper) conversation 79 (37.3%); poor 4 (1.9%). Quality of ICU discharge medicines reconciliation: Optimal

					Ward medication continuity prescription drafted and advice provided					119 (67.2%); no (proper) conversation 4 (2.3%); poor quality 1 (0.6%)
**Buckley 2015** [55]	Clinical pharmacist-managed SUP programme	Clinical pharmacist-led intervention can optimise use of SUP and help prevent inappropriate prescribing of SUP	Pharmacists with prescriptive authority for SUP medication with a defined institutional protocol using e-prescribing system with medical staff review and authorisation.	Clinical pharmacists with physician authorisation	Used electronic prescribing system	Hospital-wide including ICU	No information	No information	No information	No information
**Coon 2015** [56]	ICU transfer checklist	Incorporating a standardised checklist into existing transfer documentation would decrease the rate of inaccurate medicines reconciliation by transferring physicians and would reduce unnecessary urinary catheter	Discharge checklist inserted into electronic transfer note. ICU transfer checklist composed of sections on: Medication Reconciliation, Urinary Catheter, (venous thromboembol	ICU medical staff	Electronic insertion of checklist into transfer note	ICU (Neurosciences)	Once, on ICU transfer	None once checklist tested	None once checklist tested	The checklist compliance rate was 93% (122/131). Transition to palliative care and transfer to a non-neurologic hospital service were the most

		use, ICU readmission, length of stay, and adverse events. The standardised documentation would be valued by both transferring and accepting physicians	ism) Prophylaxis, Vitals/ Cares, Consults, and Follow-Up							common factors in non-compliance
**D'Angelo 2019** [57]	Antipsychotic discontinuation bundle	An antipsychotic discontinuation algorithm (guideline), supported by a bespoke education programme would provide: (1) audit and feedback data for staff to improve their knowledge of actual versus perceived practice with ICU antipsychotics for delirium, (2) identify potential/	1. Education of staff (physicians, nurse practitioner and nurses) on delirium management 2. Antipsychotic discontinuation algorithm. 3. Education for staff (physicians, nurse practitioners, nurses, and clinical pharmacists) Implementation of nonpharmacol	Research team provided education (Pharmacist and medical staff)	Multiprofessional education: Electronic module (bimonthly) & lectures on induction & twice weekly ICU teaching sessions Education. (Nurses): in-services to reach all shifts	ICU	Unclear. Bimonthly education for staff (physicians, nurse practitioners, nurses, and clinical pharmacists). (Nurses): in-services to reach all shifts. At induction for new staff.	No information	No information	Patients with an evaluable CAM-ICU score in the Before and After groups (35/140) and (24/141), respectively). Before: 65.7% of patients continued on antipsychotics despite a negative CAM-ICU for a minimum of 24 hours

		actual barriers to implementation, and (3) identify changes required to ensure implementation success. Implementation of the bundle would improve patient safety by increasing delirium screening, non-pharmacological management of delirium and reduce inappropriate antipsychotic therapy at transfer from ICU to hospital ward or home	ogical management of delirium							prior to ICU transfer, compared with 50% of patients in the After group
**Hammond 2017** [58]	Education on SUP guideline	Education of staff would improve awareness and knowledge of SUP guidelines and implementation thereof, would reduce	1. SUP guideline pocket card on SUP initiation and choice of agent.<br>2. Education on the SUP materials	Clinical pharmacist	Face to face 5-minute education session. One-off 5-minute education session in Jan 2015. Education	ICU	Once (Jan 2015) and on ICU service induction for medical staff	No information	No information	Due to scheduling constraints, some medical staff that worked night shifts during their first week in

		inappropriate use of SUP including continuation on transition from ICU			was provided individually and in small group sessions with medical staff during the first few days of their ICU service					the ICU did not receive the education at that time
**Hatch 2010** [59] **(After)**	Education on SUP guideline	Staff education, supported by audit and feedback on appropriate SUP use, would reduce inappropriate continuation of SUP at hospital discharge	Hospital SUP guidelines, supported by dissemination of previous audit and feedback results. Pocket guide. Memorandum on SUP distributed to ICU, medicine and surgery medical staff. Education of medical and pharmacy staff on the SUP guidance	Senior physicians to incorporate into training meetings for new medical residents. Pharmacist provided education to pharmacists	Memorandu m on SUP communicat ed via email. Senior physician training via induction meetings for new medical staff. Pharmacist face to face meeting once (October 2006) with education and audit and feedback of previous results	Critical care, medicine, and surgical services medical and pharmacy staff educated	Email of SUP memo. Medical staff education meetings. Pharmacist education session (one)	No information	No information	No information
**Heselma**	Medicatio	Medication	Pharmacists	Hospital	Pharmacist	Medical,	Once on	No	No	Intervention

**ns 2015** [14]	n review of patients transferre d from ICU to wards	review after transfer of ICU patients to the ward would reduce the rate and severity of drug-related problems (DRPs) patients encountered	undertook medication review on the ward within 48 hours of ICU patient transfer. They made recommendati ons to medical staff when DRPs were identified	pharmacist. There is no formal curriculum for clinical pharmacists in Belgium. The pharmacists in the study had all completed a 6-year course in hospital pharmacy	informed by an e-mail sent automaticall y to undertake a medication review of the patient upon ward transfer (within 48hrs). Patient cases were discussed in pharmacists' group meetings at regular intervals. Pharmacists' recommend ations for drug therapy changes were communicat ed (i) in person to the ward physicians in the intervention group; (ii) if	surgical or geriatric wards of 3 centres.	admission to the ward from ICU (within 48 hours)	information	information	: 298 received intervention (3 did not as were discharged) Control: 289 received control (10 did not on ethical grounds)

					physicians were not seen face to face, a telephone call was used; (iii) and if the telephone call was missed, an email was sent to the physician as a last reminder					
**Kram 2019** [60]	Electronic handover tool	A handover tool would enhance clinical pharmacist communication, review and transition continuity of antipsychotics therapy for ICU patients (for non-mental health indications), thereby potentially reducing inappropriate antipsychotic therapy in	Electronic handover tool developed and integrated into the e-prescribing system. Formalised education about ICU delirium, consensus guideline on pharmacological management of delirium. Education underpinned with pre-intervention	ICU pharmacists provided specialised pharmacy services, participated in daily ward rounds, and were responsible for clinical verification for their respective ICUs	Education was provided face to face. Electronic handover to pharmacists both within ICU care and on ICU to ward transfers via the e-prescribing system. The status of the handoff remained open until the AAP was	Handover commenced on ICU and continued onto the ward if the handover episode remained open	All clinical pharmacists reviewed electronic handoffs daily (0700-2330h) for their designated patients as part of their normal clinical activities	No information	No information	Electronic handovers were generated 66.7% (150) patients in the post-intervention group. The majority of patients (55.3%) with a discharge prescription in the postintervention group were not followed by a service-

		patients on hospital discharge	audit and feedback of antipsychotic use results		discontinued and subsequently closed by a clinical pharmacist					based pharmacist, compared to 38% in the pre-intervention group
**Medlock 2011** [61]	Electronic discharge letter for ICU patients	The discharge letter is the primary means of communication at patient discharge. Improving timely completion of discharge letters would improve discharge communication and reduce risks to patient safety	ICU discharge e-letter (to ward & GP). Policy change by ICU management team so all ICU patients to have e-letters that go with patient to the ward. Responsibility for completion of the letter automatically assigned and visible. Letter template to aid completion	ICU medical staff are responsible for finalising the letters	E-letter with electronic allocation and email reminders	ICU with electronic clinical information system	Uncompleted letters on ICU patient discharge prompts weekly email reminder for designated medical staff member assigned responsibility	No information	Improvement directive by management team in February of 2006. ICU team agreed to plan and designed the software and letter templates. The e-letter was tested in October - December 2006 with roll out on 1 January 2007	Percentage of ICU patients with a completed letter on discharge increased from 2.5% (before phase) to 80% in the 34 months after phase. By month 3, 89.9% of patients had a discharge letter completed on time
**Meena 2015** [47]	Education of medical staff on SUP	Improving medical staff knowledge of SUP in ICU patients would improve the use of SUP and reduce	Pre-rotation questionnaire followed by didactic education session on SUP for ICU medical staff (House	Didactic education sessions were conducted monthly by the critical care	Didactic education session provided for medical staff	No information	Single education session provided on monthly basis	No information	No information	No information

		inappropriate continuation, including at patient transition from ICU	staff)	pharmacist and the intensivist						
**Parsons Leigh 2020** [48]	ICU e-transfer tool	Employment of an evidence-informed ICU-specific e-transfer tool would improve completion and communication of care for ICU patients on ward transition	The final e-transfer tool had 8 sections: Visit Data, Goals of Care, Allergy and Intolerances, Diagnoses and Visit Issues, Course in ICU, Investigations, Medications, and Discharge to Home/Community. Used a combination of automated fields and free text fields	ICU medical staff (residents) used the e-transfer tool. Supported by 15-minute education session pre-use. Multiprofessional implementation team developed the e-transfer tool. Included medical staff (ICU and ward), outreach nurse, the CIS physician lead, clinical operations	e-transfer tool built into ICU clinical information system	On ICU pre-transfer	Could be modified in real time to minimise disruption in patient transfer planning	Individualised per patient	The implementation team built five iterations of the e-transfer tool before piloting	Two measures of transfer summary quality were used: timeliness and completeness of information. Documents produced with the e-transfer tool had significantly higher proportion of essential clinical information completed (median of 87.5% versus median of 62.5%)

				support staff, research staff and quality improvement lead. Undertook a heuristic evaluation with combination of human factors and clinical experts						
**Pavlov 2014** [49]	Medicines reconciliation on hospital admission and ICU discharge	Medicines reconciliation provides a more accurate pre-admission medication list reducing medication errors. Undertaking medicines reconciliation on patient admission and discharge, would reduce inappropriate continuation of SUP and bronchodilators	Medicines reconciliation including the patients/ representative with a review of previous discharge notes and local out-patient pharmacy records via a database. Pre-admission medicines entered on the e-medical record for review and approval within	Pharmacy technician compiled meds rec-entered on electronic medical record-reviewed and modified/approved by the admitting medical staff within 48 hours. Medical staff undertook	Interviewed subjects, or representatives when required, and reviewed previous discharge notes and local out-patient pharmacy records available through a local database. Medical staff review	Emergency room and ward	On admission; then prior to discharge or in-hospital transfers	No information	No information	No information

		on hospital discharge	48 hours. On hospital transfer a review of pre-admission and in-patient medication lists was undertaken with the patient. Finally, the medication list was added to the patient discharge summary	review of pre-admission and inpatient medication created by a discharge nurse, with the patient on ICU transfer	of pre-admission and inpatient medication lists with patient					
**Pronovost 2003** [50]	Medicines reconciliation on ICU discharge	Medicines reconciliation in ICU discharge reduces medication errors	Standardised paper medicines reconciliation forms. All ICU nurses were educated on use of the discharge survey which was available on the front of every admissions chart. Instructions on completions were also included in the research	ICU nurses completed the medicines reconciliation forms made available by ICU ward clerks	Completion of a discharge survey that identified specific types of possible medication errors that prompted discussion with an ICU physician to resolve if needed	ICU discharge	Once, prior to ICU discharge	No information	Initial staff resistance to completion of the medicines reconciliation discharge survey as 1) too time consuming; 2) it was not their responsibility to monitor medications outside the ICU; 3) it was difficult to obtain an	Compliance with the medication reconciliation process per week varied from <40% to 100% initially stabilising around mid-90%

			team's data collection spreadsheet						accurate list of prehospital medication. The discharge survey was revised so that all pre-admission, inpatient and discharge medicines were listed. Paper forms were eventually converted to electronic on the ICU clinical information system after 48 weeks	
**Stuart 2020** [53]	Antipsychotic de-escalation protocol	Pharmacist-led protocol would increase the effectiveness of discontinuation of antipsychotics for ICU delirium and reduce the inappropriate continuation at	Antipsychotic de-escalation guideline for resolved ICU delirium support by education for ICU and ward-based pharmacists. A collaborative	Pharmacists (ICU and hospital ward (internal medicine))	Pharmacists were trained on the use of the discontinuation protocol in in-service training sessions. Pharmacists	ICUs (ICU patients directly discharged from hospital) and internal medicine hospital wards	ICU pharmacists attend daily multiprofessional ward rounds (Monday-Friday)	No information	No information	Pharmacist electronic communication initiated in 52/79 (65.8%) of eligible patients in the After group

		patient hospital discharge	practice agreement enabled pharmacists (ICU and hospital ward) authority to discontinue or taper antipsychotics in ICU patients with delirium that had resolved		had authority to discontinue or taper antipsychotics in ICU patients with resolved delirium supported by the de-escalation guideline					
**Tasaka 2014** [51]	Interprofessional bundle to reduce the overutilisation of SUP	Guideline and education would inform SUP practice, reducing inappropriate continuation. Pharmacist-led intervention would improve the quality of SUP review and utilisation	The SUP guideline was promoted by publication in hospital newsletters, emails to medical staff, development of facilitator guides to use during teaching rounds and presentation to various clinician groups. Education targeted at surgery, medicine and anaesthesia	Multiprofessional team (pharmacists, physicians, nurses, and dieticians) planned and developed a bundled approach to reduce the overutilisation of SUP in adult ICU patients. ICU pharmacists undertook SUP medication reviews as	Pharmacist-led SUP intervention on the ICU with recommendations for medical staff on SUP therapy	ICU pharmacist SUP recommendations made in person during their patient rounds to the ICU medical staff, or made via text page or phone call	No information	No information	Educational effort has been streamlined to a 30-minute monthly lecture for residents rotating through the ICU	No information

			medical teams, dieticians, ICU nurses, and pharmacists. Pocket cards summarised the SUP guideline were also disseminated to ICU medical, pharmacy staff. Training sessions were repeated monthly to improve awareness of appropriate SUP use	part of their daily rounds and made recommendations to ICU medical staff on actions						
**Zeigler 2008** [52]	Medicines reconciliation	Medicines reconciliation on admission and at patient transition interfaces, would decrease the incidence of medication errors	Medicines reconciliation consisted of a medication history entered into the e-health record, reviewed by the admitting physician. Upon level of care transfers (eg, ICU to non-ICU unit) medication profiles are	Pharmacists and nurses undertook the medicines reconciliation with medical staff review at each transfer point	Medicines reconciliation on individual patient basis. Upon level of care transfer (e.g., ICU to non-ICU unit) or hospital discharge, medication profiles are printed and	On admission and upon level of care transfer (e.g., ICU to non-ICU unit) or hospital discharge	On admission and upon each level of care transfer	No information	No information	No information

			printed and reviewed by the primary physician. Prior to implementation of medicines reconciliation, education of clinical staff (medical, nursing, pharmacy) on process and roles was completed. Education was done by classes, a Web-based training module, presentations at hospital committee meetings, and one-to-one communication		reviewed by the lead physician, and existing agents are ordered to be either discontinued or resumed.					

Table S1: Summary of intervention details using TIDieR template [41]

DRPs: Drug-related problems; ICU: Intensive care unit; SUP: Stress ulceration prophylaxis
