# Supplementary material for: Medication-related interventions to improve medication safety and patient outcomes on transition from adult intensive care settings: a systematic review and meta-analysis
Source: BMJ Qual Saf. 2022 Jan 18;31(8):609–22. doi: 10.1136/bmjqs-2021-013760 (PMC9304084; doi:10.1136/bmjqs-2021-013760)
Supplement: Supplementary data [file bmjqs-2021-013760supp003.pdf]

Additional File 3: Economic outcomes and Summary of findings

Economic Evaluations

Four studies included an economic evaluation (Table S4) (13,54,55,59). However, only three of the studies included details for ICU patients on the interface of ward transfer (13,54,59). Two studies focused on the cost avoidance of reducing inappropriate continuation of SUP (54,59). Bosma et al (13), calculated the cost-benefit of the pharmacist-led medicines reconciliation programme in their two-centre Dutch study. They reported a positive cost–benefit ratio of 2.48, indicating a potential net cost–benefit of 2018 €103 per patient based on intervention costs and pADEs prevented.

Author/ Year/ Country	Medication Outcome(s)	Methods used to identify medication outcome(s)	Patient Outcomes	Economic Evaluation
**Anstey 2019** [54] Australia	Inappropriate stress ulcer prophylaxis (SUP) continuation [Hospital discharge].<br>Before: 78/184 (42.4%) versus (Vs.) After: 11/143 (7.7%) p<0.001	Standardised data extraction form of patients on SUP without an indication (from evidence-based SUP indication list) [prospective chart review completed by medical staff not involved in prescribing of SUP]	No between group comparison of gastro-intestinal (GI) bleed, pneumonia or Clostridium difficile rates	SUP deprescribing data extrapolated to pan-Australia. Based on 2016 data, the additional lifetime cost (assuming 10-year endurance) of inappropriate SUP continuation post-ICU in a year is AUD $20.82 million. Under the shorter scenarios of two- or five-year continuation, this figure reduces proportionally to AUD $4.16 million and AUD $10.41 million, respectively
**Bosma 2018** [13] Netherla nds	Medication Errors (MEs) on transfer [ICU discharge].<br>Before: 73.9% of 203 patients had ≥1 MEs Vs. After: 41.2% of 177 patients. A reduction of 44.2%. Odds Ratio adjusted (OR_{adj}) 0.24 [95% CI 0.15–0.37], adjusted for severity of illness	ME at discharge was an unintentional discrepancy between the actual patient medication chart compared to the best possible general ward medication list (24 hours after the ICU discharge). When possible, this included a ward physician discussion [completed by two ICU pharmacists with crosschecking of data]. All MEs were validated as part of the potential adverse drug events (pADE) assessment. All MEs were randomly assigned and assessed by two ICU healthcare professionals independently, reaching consensus when required	pADE on transfer.<br>Before: Proportion of patients with a pADE ≥ 0.01 was 69.5% of 203 patients Vs. 36.2% of 177 patients, a reduction of 47.9%. OR_{adj} 0.26 [95% CI 0.17–0.40] adjusted for severity of illness	Positive cost–benefit ratio = 2.48, indicating a potential net cost–benefit of €103 per patient. Costs of the intervention were € 7476 at admission and €7256 at discharge. At admission 7.33 pADEs were prevented, leading to a cost avoidance of €7911 at admission. At discharge 26.59 pADEs were prevented, leading to a cost avoidance of €28,687. The cost–benefit remained positive in the sensitivity analysis
**Buckley 2015** [55] USA	Inappropriate SUP continuation [ICU discharge].<br>Before: 67.8% (118/174) patients Vs. After: 38.9% (65/167) patients, p<0.001	SUP was considered inappropriate in ICU patients without any major risk factors from a standardised list or pre-admission therapy. SUP appropriateness assessed retrospectively by research team chart review	No between group comparison of upper GI bleed, pneumonia or *Clostridium difficile* rates	ICU and ward SUP costs were compared in Before and After periods, but these did not specifically relate to ICU patient hospital discharge data
**Coon 2015** [56]	Patient transfers with active IV antihypertensives or	Medication reconciliation (med rec) of intravenous (IV) antihypertensives and	No difference in mean length of stay (LOS) on hospital ward after	None

USA	vasopressors (surrogate marker of medicines reconciliation) [ICU discharge]. Before: 36.2% (47/130) of patients Vs. After: 9.9% (13/131), p=0.001	vasopressors was prospectively assessed. Med rec was deemed not undertaken if the e-prescribing system had an active prescription for either IV therapy groups on ICU discharge	ICU transfer (5 days in both groups, p= 0.31). No between group difference (Before Vs. After) in adverse events (as measured by ICU readmissions (4(3) Vs. 5(4); p=0.74) or rapid response team calls (2(2) Vs. 4(3); p=0.69))	
**D'Angelo 2019** [57] USA	Inappropriate antipsychotic continuation [ICU discharge]. After: OR 0.47 [95%CI 0.26-0.86] Antipsychotic discontinuation [72hrs after ICU discharge]. Before: 35.9% of 140 patients Vs. After: 61.5% of 141 patients. ORadj: 4.55 [95% CI: 1.44-14.43]. Inappropriate antipsychotic continuation [Hospital Discharge]. Before: 15.7% of 140 patients Vs. After: 8.5% of 141 patients. ORadj: 0.4 [95% CI 0.18-0.89]	Retrospective data collection from the patient medical chart review including delirium status at set time periods. Antipsychotic medication exposure was collected for each patient. Antipsychotics were recommended to be stopped once the patient was delirium-free for 48 hours	No between group comparison of hospital LOS of ICU transfer patients	None
**Hammond 2017** [58] USA	Inappropriate SUP continuation [ICU discharge]. Before: 60% (61/101) patients Vs. After: 53.4% (63/118) patients, p=0.297 Inappropriate SUP continuation [Hospital discharge]. Before: 17.8% (18/101) patients Vs. After: 13.6% (16/118) patients, p=0.368	Appropriateness of SUP was assessed by chart review at the time of transfer from the ICU. Assessment was against set guideline criteria for SUP clinical appropriateness.	No difference in adverse events related to SUP between the intervention periods. E.g., pneumonia, 5(5%) before vs. 6(5%) after; p>0.99	None
**Wohlt 2007** [62] **(Before) Hatch**	Inappropriate SUP continuation [ICU discharge]. Before: 48% (189/394) patients Vs. After: 23.6% (84/356)	Retrospective review of patient electronic medical records, pharmacy systems and discharge records. Assessment of SUP appropriateness against approved local	None	Single ICU data indicated the reduction in inappropriate SUP drug use by 64.3% (After), leading to over USD $200,000

**2010** [59] **(After)** USA	patients<br>Inappropriate SUP continuation [Hospital discharge].<br>Before: 24.4% (96/394) patients Vs. After: 8.7% (31/356) patients	guidelines. Standardised collection form used.		(2010) in estimated 1-year drug cost savings
**Heselmans 2015** [14] Belgium	Incidence of drug-related problems (DRPs). Ward stay within 48hrs of ICU transfer.<br>Intervention: 54.1% (203/375) DRPs were adjusted on time Vs. Control: 12.8% (47/368).<br>OR_{adj} 15.6 [95%CI 9.4–25.9] after adjustment for differences in types of DRPs between the groups.<br>Intervention effect by clinical impact category of DRPs.<br>Major (n=184): 11.3 [95%CI 4.9–25.4]; Moderate (n=97): 19.6 [95%CI 5.9–64.4]; Minor (n=396): 14.1 [95%CI 6.9–28.6]; None (n=66): 0.9 [95%CI 0.2–3.1]	Pharmacists used standardised data collection form to record DRPs identified. A minimum of one pharmacist reviewed each patient's medical records and completed data collection. Patient cases were also discussed at regular group pharmacist meetings. DRPs and pharmacist interventions based on the French Society of Clinical Pharmacy scheme classification. The clinical impact of the DRPs was assessed by a panel of 8 internists individually using an adapted version of The National Coordinating Council for Medication Error Reporting and Prevention (NCC MERP). Any differences in ratings were discussed and resolved by discussion	No differences in any of the patient outcomes in Intervention Vs. Control groups.<br>Hospital discharge mortality rates, 22 Vs. 22.<br>ICU re-admission rates, 72 Vs. 72.<br>Hospital LOS (mean 34.2 days [95%CI 29.6–38.9] Vs. mean 34.5 days [95%CI 30.0–38.9]	None
**Kram 2019** [60] USA	Inappropriate antipsychotic continuation [Hospital discharge].<br>Before: 19.5% (26/133) of patients Vs. After: 11.6% (26/225) of patients	Atypical antipsychotic prescription on discharge was deemed inappropriate (by consensus criteria) if the patient was documented at their baseline mental status in the medical record, or if there was no documented indication for continuation of antipsychotic therapy. Data collected by pharmacists from the electronic prescribing system.	No differences in Before and After periods for median (IQR) ICU LOS (12.86 (5.07-21.78) Vs. 14.72 (6.33-23.65)) or median (IQR) hospital LOS (24.71 (14.74-37.31) VS. 28.24 (16.30-42.28)) days	None
**Medlock 2011** [61] Netherlands	Completion of ICU discharge letter (including medication information) [ICU discharge].<br>Before: 2.5% of 1872 patients Vs. After: 80% of 4951 patients.	Data on electronic letter completion were taken from the patient data management system (PDMS). Dictated letters data were collected from matching the patient PDMS and hospital letters databases.	No difference in patient mortality rate between the before and after groups (17.5 Vs. 17.8%; p=0.74)	None

	Patients with a finalised ICU discharge letter. Before: 11.4% of 1872 patients Vs. After: 96.6% of 4951 patients.<br>Time to finalise ICU Discharge letter.<br>Before: median (IQR) 23 (9-41) days Vs. median (IQR) 4 (2-9) days, p<0.0001			
**Meena 2015** [47] USA	Inappropriate SUP continuation [ICU discharge].<br>Before: 68.7% (68/99) of patients Vs. After: 36.5% (42/115) of patients, p<0.001.<br>Inappropriate SUP continuation [Hospital Discharge].<br>Before: 23.9% (22/92) patients vs. After: 16.5% (18/109) of patients, p=0.19	Retrospective chart review by research team. Inappropriate SUP defined by not meeting local guidelines requiring at least one major or minor SUP indication.	None	None
**Parsons Leigh 2020** [48] Canada	Transfer documentation of active medications [ICU discharge].<br>Before (dictation): 80% (24/30) Vs. After (electronic (e) transfer tool): 97% (29/30) patients, p=0.044<br>Transfer documentation of medicines reconciliation.<br>Before (dictation): 27% (8/30) Vs. After (etransfer tool): 53% (16/30) patients, p=0.035	Standardised data collection form capturing completion rates of 8 essential transfer elements (including active medications and medicines reconciliation). Binary score, either present or absent for dictated and etransfer tools.	None	None
**Pavlov 2014** [49] USA	Inappropriate SUP continuation [ICU discharge].<br>Before Intervention OR_{adj} 2.5 [95%CI 1.4–4.7]<br>Inappropriate bronchodilator	Data extracted from patient medical records. Medication data collated from the dictated admission and discharge notes (Before) or pharmacy technician/ medical staff pre-admission list and discharge list from the nurse	ICU patient mortality rate was lower in the After group compared to Before group 13.2 vs. 20.6%, p=0.006. However, mortality rate not clearly linked to the	None

	continuation.<br>Before Intervention ORadj 2.4 [95%CI 0.98–5.9].<br>Inappropriate continuation of Either (SUP or bronchodilator).<br>Before 46/253 (18.2%); After 24/291 (8.2%), p=0.006	derived clinical summary (After). Case notes of patients discharged on SUP or bronchodilators were reviewed to confirm if any clinical indication for treatment to continue	intervention - causality	
**Pronovost 2003** [50]<br>USA	Evaluated discharge prescriptions with MEs/week [ICU discharge].<br>Before: 94% (31/33) in 2-week baseline Vs. After: average 5% per week over 22 weeks	Standardised data collection tool completed by ICU nurses. Nurses reviewed the patients ICU medical record and medication prescriptions on ICU discharge. Potential MEs identified via 3 basic prompts with confirmation of intended changes with medical staff. Nurses conformed pre-admission medication and allergy status with the patient as well. An ME was defined as a prescription change as a result of this process	None	None
**Stuart 2020** [53]<br>USA	Inappropriate antipsychotic continuation [ICU discharge]<br>Before: 35% (21/60) Vs. After: 35.9% (23/64) of patients, p=0.913<br>Inappropriate antipsychotic continuation [Hospital discharge]<br>Before: 32.9% (26/79) Vs. After: 7.6% (6/79) of patients, p<0.001	All data collected using a standardised case report form retrospectively. For assessment of the primary outcome (inappropriate continuation of antipsychotics at hospital discharge), obtained by the primary author via retrospective patient record review.	No differences in between group comparison in median (IQR) ICU LOS (Before 14 (8,28) Vs. 10 (7,23) days; p=0.1) or hospital LOS (Before 25 (13,34) Vs. After 19 (13,30) days; p=0.055)	None
**Tasaka 2014** [51]<br>USA	Inappropriate SUP continuation [ICU discharge].<br>Before (Post-CPOE); 8% 6/74 Vs. After: 4% (2/50), p=0.54.<br>Inappropriate SUP continuation [Hospital discharge].<br>Before (Post-CPOE); 7% (5/73) Vs. After: 0% (0/44), p=0.22	Data collected retrospectively from a review of patient electronic medical and medication records [by research team pharmacist]	None	None
**Zeigler 2008** [52]	Inappropriate SUP continuation [ICU discharge].	Electronic admission report used to identify all patients admitted to the ICUs and receiving SUP.	None	None

USA	Before: 85% (45/53) of patients Vs. After: 79% (48/61) of patients, p=0.393).<br>Inappropriate SUP continuation [Hospital discharge].<br>Before: 14% (6/44) of patients Vs. After: 23% (10/43) of patients, p=0.247	Med rec data available from the electronic medical record. SUP was considered inappropriate if the patients did not have at least 1 major risk factor or 2 minor risk factors from a locally agreed guideline.		

Table S2: Summary of study findings and methods used to identify medication outcome(s)

DRPs: Drug-related problems; ICU: Intensive care unit; etransfer: Electronic transfer; GI: Gastro-Intestinal; LOS: Length of stay; MEs: medication Errors; pADEs: OR_{adj}: Odds Ratio – adjusted; pADEs: Potential Adverse Events; SUP: Stress Ulceration prophylaxis; Vs.: Versus.
