# Supplementary material for: Medication-related interventions to improve medication safety and patient outcomes on transition from adult intensive care settings: a systematic review and meta-analysis
Source: BMJ Qual Saf. 2022 Jan 18;31(8):609–22. doi: 10.1136/bmjqs-2021-013760 (PMC9304084; doi:10.1136/bmjqs-2021-013760)
Supplement: Supplementary data [file bmjqs-2021-013760supp004.pdf]

Additional File 4: Risk of Bias and GRADE assessments

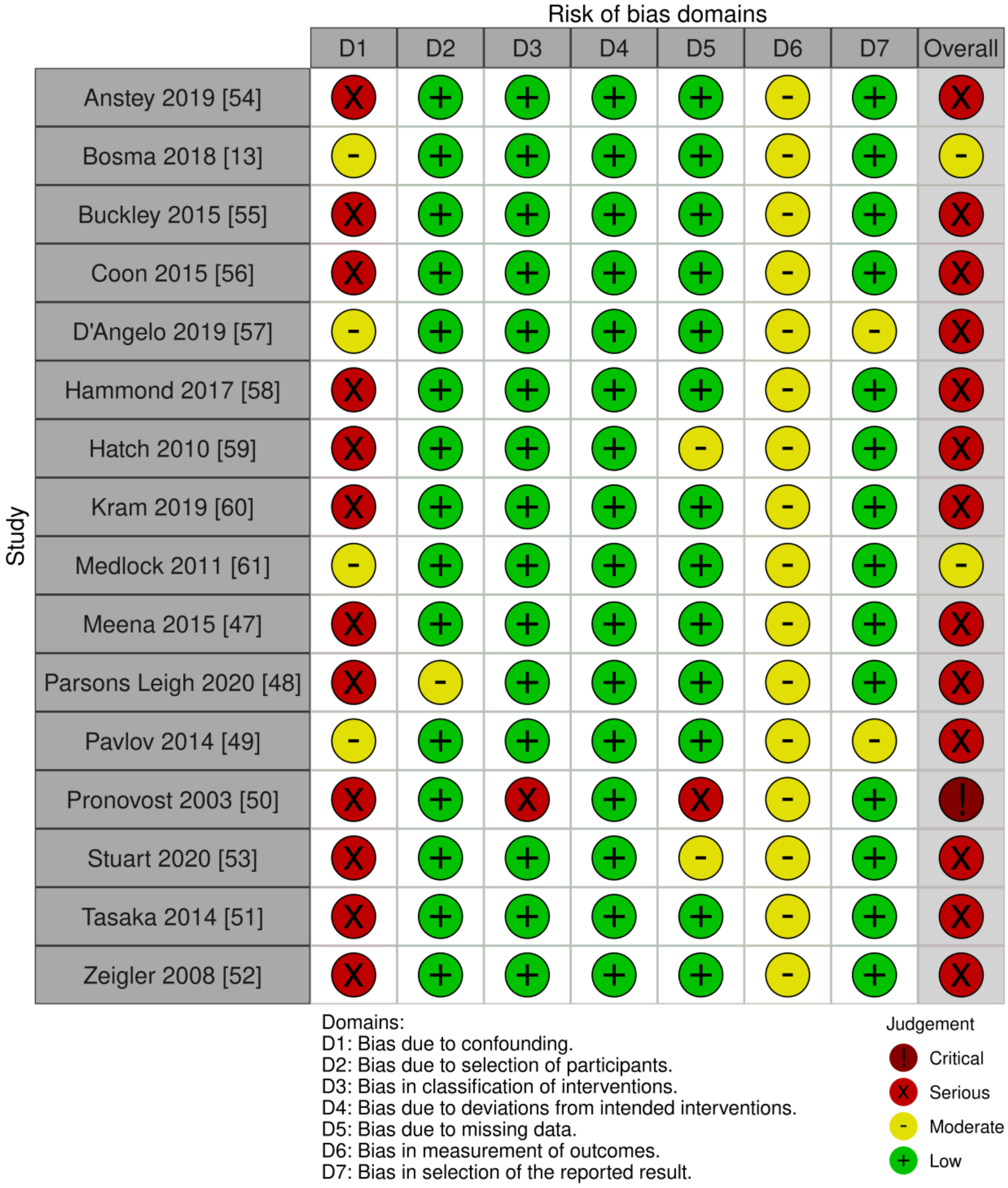

Figure S1. Robvis representation of ROBINS-I risk of bias assessments of non-randomised controlled trial studies

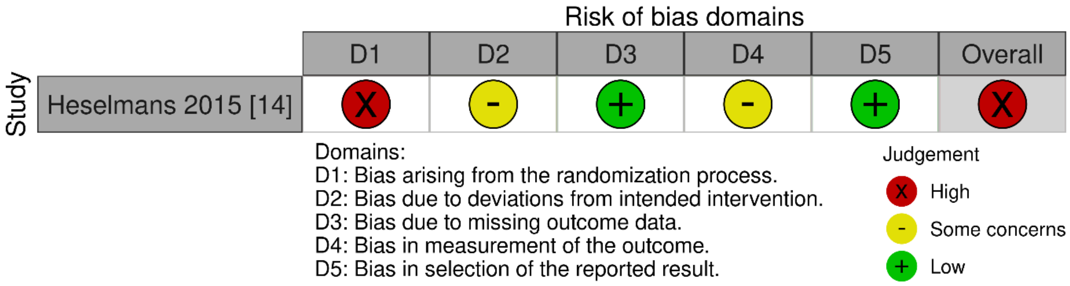

Figure S2. Robvis representation of ROB2.0 risk of bias assessments of the randomised controlled trial study

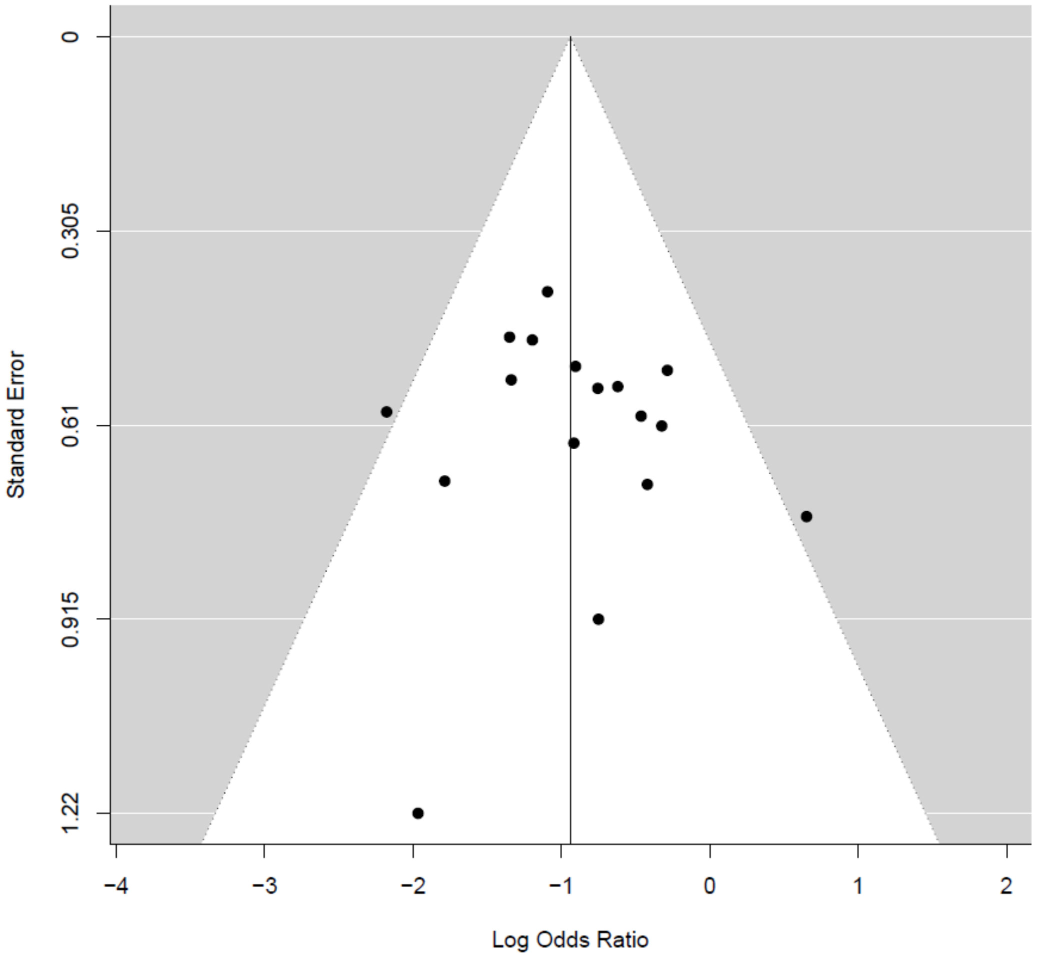

Figure S3. Funnel plot of the treatment effect estimates from individual studies included in the meta-analysis

Certainty assessment							Effect	Certainty
№ of studies	Study design	Risk of bias	Inconsistency	Indirectness	Imprecision	Other considerations	Odds Ratio (OR) (95% CI)	
Main meta-analysis. All de-escalation studies - ICU discharge inappropriate medication therapy								
9	observational studies	very serious[a]	not serious	serious[b]	not serious	strong association[c]	OR 0.45 (0.31 to 0.63)	⊕⊕◯◯ LOW
Main meta-analysis. All de-escalation studies - Hospital discharge inappropriate medication therapy								
9	observational studies	very serious[a]	serious[d]	serious[b]	not serious	strong association[c]	OR 0.39 (0.2 to 0.76)	⊕◯◯◯ VERY LOW
Sub-group meta-analysis. Multicomponent de-escalation studies - ICU discharge inappropriate medication therapy								
3	observational studies	very serious[a]	not serious	not serious	not serious	strong association[c]	OR 0.37 (0.23 to 0.59)	⊕⊕⊕◯ MODERATE
Subgroup meta-analysis. Multicomponent de-escalation studies - Hospital discharge inappropriate medication therapy								
6	observational studies	very serious[a]	not serious	not serious	not serious	strong association[c]	OR 0.26 (0.13 to 0.55)	⊕⊕⊕◯ MODERATE
Subgroup meta-analysis. Single component de-escalation studies - ICU discharge inappropriate medication therapy								
5	observational studies	very serious[a]	not serious	serious[b]	not serious	strong associationc	OR 0.42 (0.24 to 0.74)	⊕⊕◯◯ LOW
Subgroup meta-analysis. Single component de-escalation studies - Hospital discharge inappropriate medication therapy								
3	observational studies	very serious[a]	not serious	serious[b]	not serious	None	OR 0.77 (0.16 to 3.74)	⊕◯◯◯ VERY LOW

Table S3: Meta-analysis GRADE assessments

Explanations

[a]High-risk of bias on ROBINS-I (downgraded 2 levels)

[b]Significant variability in intervention components and timing of delivery (downgraded 1 level)

[c]Large effect – OR <0.5 (upgraded 1 level)

[d]Heterogeneity (I^2 ≥75%) (downgraded 1 level)
